# Uncertainty Quantification of Linear Scaling, Machine Learning, and Density Functional Theory Derived Thermodynamics for the Catalytic Partial Oxidation of Methane on Rhodium

**DOI:** 10.1021/acs.jpcc.4c05107

**Published:** 2024-10-03

**Authors:** Christopher J. Blais, Chao Xu, Richard H. West

**Affiliations:** Department of Chemical Engineering, Northeastern University, Boston, Massachusetts 02115, United States

## Abstract

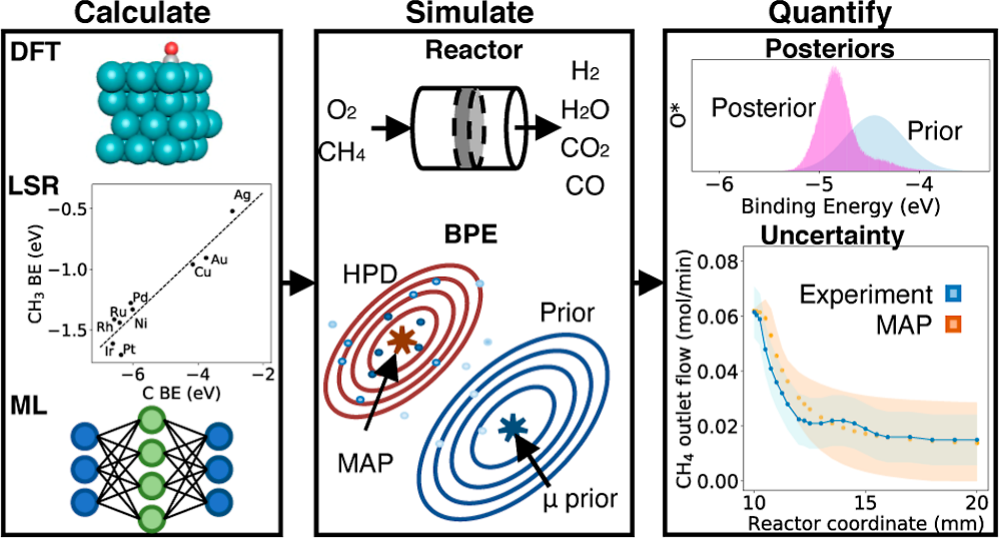

Accurate and complete microkinetic models (MKMs) are powerful for anticipating the behavior of complex chemical systems at different operating conditions. In heterogeneous catalysis, they can be further used for the rapid development and screening of new catalysts. Density functional theory (DFT) is often used to calculate the parameters used in MKMs with relatively high fidelity. However, given the high cost of DFT calculations for adsorbates in heterogeneous catalysis, linear scaling relations (LSRs) and machine learning (ML) models were developed to give rapid estimates of the parameters in MKM. Regardless of the method, few studies have attempted to quantify the uncertainty in catalytic MKMs, as the uncertainties are often orders of magnitude larger than those for gas phase models. This study explores uncertainty quantification and Bayesian Parameter Estimation for thermodynamic parameters calculated by DFT, LSRs, and GemNet-OC, a ML model developed under the Open Catalyst Project. A model for catalytic partial oxidation of methane (CPOX) on Rhodium was chosen as a case study, in which the model’s thermodynamic parameters and their associated uncertainties were determined using DFT, LSR, and GemNet-OC. Markov Chain Monte Carlo coupled with Ensemble Slice Sampling was used to sample the highest probability density (HPD) region of the posterior and determine the maximum of the a posteriori (MAP) for each thermodynamic parameter included. The optimized microkinetic models for each of the three estimation methods had quite similar mechanisms and agreed well with the experimental data for gas phase mole fractions. Exploration of the HPD region of the posterior further revealed that adsorbed hydroxide and oxygen likely bind on facets other than Rhodium 111. The demonstrated workflow addresses the issue of inaccuracies arising from the integration of data from multiple sources by considering both experimental and computational uncertainties, and further reveals information about the active site that would not have been discovered without considering the posterior.

## 1 Introduction

The significance of heterogeneous catalysis extends across fields such as energy production,^1^ carbon dioxide conversion,^2,3^ and the synthesis of numerous chemicals,^4,5^ underlining its indispensable role in advancing sustainable and economically viable chemical processes.^6,7^ Accurate chemical models are important, especially in the context of catalyst screening and discovery, where having an accurate model prior to synthesizing a new catalyst could save time, money, and resources. Mean field microkinetic modeling (MKM) is a valuable tool to quantitatively describe the reaction rates and intermediates’ thermodynamics of a process, as it is less computationally taxing compared to spatially resolved methods like Kinetic Monte Carlo.^8,9^ Therefore, MKM is often used for process design^10^ and theoretical catalyst screening.^11,12^

To determine thermodynamics in MKM, Density Functional Theory (DFT) is a widely acknowledged quantum mechanical approach to study the energy of intermediates in a model. However, its computational expense for solid-state matter poses a challenge. Surrogates such as linear scaling relationships (LSRs)^13−15^ and machine learning (ML) models^16,17^ are developed to approximate the DFT results at much lower cost. LSRs connect the molecular binding energy to the binding energy of the atom(s) in a molecule that are bonded to the surface. They can be used to estimate the difference in adsorption energy of a molecule between two metal surfaces. In the context of MKM, LSRs help provide energy estimates for intermediates on different surfaces, so the material search space can be expanded for catalyst discovery. Previous studies show that LSRs can be applied in modeling many commercially important systems such as synthesis gas conversion,^18^ oxygen reduction,^19^ etc. However, LSRs are sometimes limited by the morphology of catalysts and the coverage of the adsorbates, and they appear to fail on some alloy systems due to the site specificity and lateral interactions.^15^ In addition, utilizing LSRs still requires conducting a few DFT calculations on similar adsorption systems to establish the linear relationship. It should be noted that the linear scaling relationships referenced throughout this article refer to the linear relationships between adsorbate binding energies, not the linear relationship between the activation energy of a reaction and the enthalpy of reaction.

Given the limitation of LSRs, ML models have been developed to estimate the molecular or atomic energy in a wider range of materials. There are various ML models to help accelerate heterogeneous catalysis modeling.^20^ This includes ML-aided potential energy surface construction,^21,22^ atomistic structure and potential estimation,^23−25^ and material designs (finding the optimal composition of materials).^26,27^ Both kinetics and thermodynamics evaluations in MKM can substantially benefit from ML models. In this study, ML-predicted thermodynamics are used. A neural network named GemNet-OC,^28^ developed under Open Catalyst Project (OCP),^16^ was picked to carry out the task. GemNet-OC has been demonstrated to have good accuracy for energy and force estimation, and has been used in several works.^29,30^

DFT, LSRs, and ML models all have uncertainty associated with their predictions of species properties, which can be propagated forward through a MKM to reveal the uncertainty in quantities of interest such as turnover frequency, conversion, selectivity, etc. Previous studies have highlighted the importance of propagating energetic parameter uncertainties to industrial operating conditions.^30−32^ Beyond simply determining a “most probable” mechanism, analyzing possible reaction pathways within a given uncertainty space can reveal entirely different reaction pathways and active sites.^33−36^

Bayes theorem proposes a method for incorporating the prior uncertainties of a model and the marginal probability of observed data as a means of generating a most probable set of parameters. The process for obtaining these credible values is referred to as Bayesian Parameter Estimation (BPE). BPE can also be used to generate the highest probability density (HPD) region around the most likely parameters, which in essence is a probability distribution that has been informed both by prior knowledge and experimental data. This requires comprehensively exploring the parameter space within the model, which can be difficult for a complex system with a large number of parameters. Various methods can be used to efficiently explore the uncertainty space, including surrogate models like polynomial chaos expansion,^37^ grid based approaches,^38^ and Monte Carlo simulation based methods.^35^ Monte Carlo methods are expensive, but they are applicable to almost any model, and can be made more efficient through sophisticated sampling methods.^39^ Markov Chain Monte Carlo (MCMC) is particularly useful for model uncertainty quantification, because it implements a probability based approach to sampling, where the “jump” to the next sampled point is based on the relative probability of the previous point. BPE coupled with MCMC has been used successfully for surface chemistry models already in several studies, proving that it is a robust, albeit expensive, method for generating posteriors for catalytic systems.^35,36^

In this study, BPE was performed via MCMC sampling to reveal posterior uncertainties in both the input species binding energies and the output molar flow rates. Quantifying the uncertainties in models generated using different thermodynamic calculation method (DFT, LSRs, and GemNet model) provided a more informed basis for comparing them than simply analyzing the initial, unoptimized models.

This study establishes a workflow to optimize the model according to experimental and computational uncertainties. Data from varied sources are often incorporated in MKMs to reduce the number of DFT calculations or experimental values required, but this integration can introduce inaccuracies and inconsistencies. The proposed workflow improves the coherence of data calculated through different techniques, yielding results that align more closely with experimental observations than simply amalgamating the data without modification. The findings also illustrate that BPE serves as a valuable tool for pinpointing species with inaccurate thermodynamics, paving the way for subsequent fine-tuning through DFT.

## 2 Methods

### 2.1 Microkinetic Model

The microkinetic model used was adapted from the original model developed by Mazeau et al.^12,40^ This model was constructed using the Reaction Mechanism Generator (RMG),^41^ a Python-based tool for automatically constructing microkinetic models. Briefly, RMG uses a decision tree algorithm for both thermodynamic and kinetic parameter estimation, in addition to a substantial database of experimentally and computationally derived values. An input file specifies all of the known reactants, products, and intermediates, which are then allowed to react together and create new species, adding species into the “core” model based on their generation rates. Activation energies and pre-exponential factors are estimated using rules for reactions with similarly structured reactants. The activation energy is scaled in these rules using the enthalpy of reaction via Brønstead–Evans–Polanyi (BEP) relationships.

The RMG-generated model has 19 gas-phase species, 13 adsorbates, and 80 elementary reactions. Of the 13 adsorbates, CH_4_*, H_2_O*, CO_2_*, H_2_* were considered to be physisorbed, which required treatment as a 2D gas for partition function calculations in Section 2.2. The complete mechanism is also included in this paper’s Supporting Information as a Cantera input YAML file.

Following the adjustments of thermodynamic parameters according to DFT, LSRs, and GemNet, the kinetics in the model were adjusted to account for the change in the activation energies as the adsorbate enthalpies of formation were varied. A well-accepted method for this is to use BEP relations to linearly adjust the activation energy for a given reaction based on the enthalpy change of the reaction. Doing this after the intermediates’ energies are estimated creates a dynamic MKM where changes in the species’ thermodynamics have a realistic effect on the activation energies. Unfortunately, the data for BEP relationships is often scarce for catalytic reactions because it relies on the calculation of multiple transition states, which can be a lengthy and expensive process for surface systems. Instead, this study used the Blowers-Masel approximation (BMA) to modify the reaction barriers,^42^ as shown in eqs 1 and 2 below. The derivation of BMA parameters only needs the activation energy and reaction enthalpy of one reaction, circumventing issues related to data sparsity. A previous study has successfully implemented BMA in heterogeneous catalysis modeling.^40^

The model from Mazeau et al. was modified so that all of the surface reaction used Blowers-Masel relationships for dynamically calculating the activation energy,^42^ instead of using a static activation energy
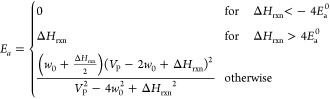
1where
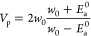
2*E*_a_^0^ is the intrinsic energy and equals the activation energy when Δ*H*_rxn_ = 0, and *w*_0_ is a parameter that, in the original derivation, represents the average of the bond dissociation energy of the broken bond and the bond being formed. Xu et al. showed that the activation energy *Ea* is highly insensitive to *w*_0_, so the only parameter that needs to be derived is *E*_a_^0^.

The parameters used in the BPE were the enthalpies of formation for each surface species. Defining the relationship between the activation energy and the enthalpy of each reaction allowed the model to realistically change the kinetics of the microkinetic model. A complete BPE analysis could in theory include all of the parameters in the model, including the *E*_a_ and pre-exponential factor for each reaction. However, an analysis of this scale would be both computationally expensive and not useful for the task at hand, namely comparing the associated uncertainty for thermodynamic estimation methods.

### 2.2 Species Thermodynamics

#### 2.2.1 DFT Calculations

DFT calculations were performed in Quantum Espresso (QE) version 7.0^43,44^ with SG15 Optimized Norm-Conserving Vanderbilt pseudopotentials^45^ for 13 species on Rh(111) and 2 species on Rh(211). A complete list of these species and their prior uncertainties can be found in Table 1. The BEEF-vdW functional was used for structure relaxations and uncertainty quantification.^46^ The molecular structures were constructed with Atomic Simulation Environment (ASE).^47^ Equation of state^48^ was used to determine the lattice constant of the Rh cell with a wave function kinetic energy cutoff of 60 Ry and a well-converged Monkhorst–Pack mesh of (15 × 15 × 15). The electron orbitals were broadened using the Mazari-Vanderbilt smearing method with the value of 0.01 Ry. The lattice constant was estimated as 3.85 Å, aligning well with the literature value.^49^ 3 × 3 × 4 Rh(111) and Rh(211) slabs were made with vacuum of 17 Å. The bottom 2 layers were fixed, and the top 2 layers in the slabs were relaxed by QE with a (5 × 5 × 1) *k*-point grid and the same energy cutoff and smearing conditions as used for the lattice constant calculation. The adsorbates’ gas-phase counterparts were relaxed in a gamma-centered 13 × 13 × 13 Å^3^ cell until the forces fell below 0.01 eV/Å. The functional, energy cutoff, and the smearing methods were the same as for the slab calculations.

**Table 1:** Prior Values of Binding Energy and 2σ Uncertainties for all Surface Species in the DFT, LSR, and ML Models

species	DFT prior (eV)	LSR prior (eV)	ML prior (eV)
H*	–2.53 ± 0.13	–2.53 ± 0.19	–2.65 ± 0.33
CO_2_*	–0.95 ± 0.25	–0.95 ± 0.25	–0.95 ± 0.25
CO*	–1.90 ± 0.29	–1.82 ± 0.54	–1.62 ± 0.42
CH_4_*	–0.10 ± 0.16	–0.10 ± 0.16	–0.10 ± 0.16
O* (111)	–4.77 ± 0.33	–4.77 ± 0.61	–4.70 ± 0.45
O* (211)	–4.73 ± 0.50	–4.73 ± 0.61	–4.74 ± 0.58
CH_2_*	–3.91 ± 0.26	–4.02 ± 0.53	–4.01 ± 0.40
CH_3_*	–1.64 ± 0.30	–1.99 ± 0.41	–1.58 ± 0.42
CH*	–6.29 ± 0.38	–6.46 ± 0.67	–6.40 ± 0.49
C*	–6.95 ± 0.42	–6.95 ± 0.87	–7.03 ± 0.51
H_2_*	–0.041 ± 0.095	–0.041 ± 0.095	–0.041 ± 0.095
OH* (111)	–2.73 ± 0.30	–2.51 ± 0.46	–2.59 ± 0.42
OH* (211)	–3.08 ± 0.52	–3.05 ± 0.49	–3.03 ± 0.60
H_2_O*	–0.19 ± 0.26	–0.19 ± 0.26	–0.19 ± 0.26
CHO*	–2.71 ± 0.33	–2.76 ± 0.44	–2.43 ± 0.44

The relaxed gas-phase molecules were then placed on the ontop, bridge, fcc and hcp sites on the (111) surface and relaxed with the OCP calculator^16^ with the pretrained GemNet-OC-L-F^28^ model until the atomic forces were under 0.05 eV/Å. All the unique sites on the (211) surfaces were identified by Pymatgen,^50^ and relaxed by the same OCP calculator. The lowest energy structures were further relaxed with QE using the same settings as the slab relaxation until the atomic forces were under 0.01 eV/Å. The vibration analyses were performed using ASE, and imaginary frequencies for physisorbed species were approximated as 12 cm^–1^ as discussed in refs (29 and 51). The Rh(211) slab and adsorbate DFT calculations used the same settings as the Rh(111) surfaces.

The microkinetic models used the NASA 7-coefficient polynomial parametrization to describe heat of formation Δ*H*, entropy Δ*S*, and temperature-dependent heat capacity *C*_p_ at low and high temperature ranges.^52^ The polynomial parameters can be determined from heat of formation at 0 K and vibrational frequencies through partition functions. The routine reported by Blondal et al.^53^ was used in this study to generate the parameters. For the cases where the first 2 frequencies are less than 100 cm^–1^, a 2D gas model was applied instead of the harmonic oscillator approximation.^54^ To calculate heat of formation at 0 K of an adsorbate, the energy of its gas-phase precursor is calculated and corrected to align with Active Thermochemical Tables (ATcT),^55^ and the zero-point corrected adsorption energy is added on top of the ATcT corrected energy of the gas-phase precursor. As reported by Klippenstein et al.,^56^ a reference reaction should be used to reduce the error introduced by different wave functions. Therefore, a similar hypothetical reaction shown in eq 3, was used to describe the heat of formation of any species formed by a combination of H, C, N, and O.
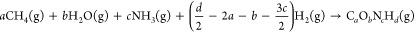
3The heat of formation of the hypothetical reaction eq 3 can be written as eq 4
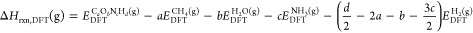
4where *E*_DFT_^species^^(g)^ is the zero-point-corrected energy of a species calculated by DFT, in this case the BEEF-vdW functional. The heat of formation of species C_*a*_O_*b*_N_*c*_H_*d*_ corrected by ATcT reference values can be calculated using eq 5.
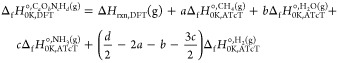
5The heat of formation of the adsorbed C_*a*_O_*b*_N_*c*_H_*d*_ is then calculated by eq 6
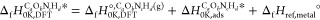
6Δ_f_*H*_ref,metal_° = 0 for rhodium and platinum because they are not included in ATcT.  is the adsorption energy of C_*a*_O_*b*_N_*c*_H_d_* which can be calculated through eq 7
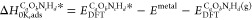
7 is the zero-point corrected energy of the adsorbed C_*a*_O_*b*_N_*c*_H_d_* calculated by DFT, *E*^metal^ is the metal slab energy, and  is the zero-point corrected energy of the gas-phase species calculated using DFT.

When these are all combined to determine , the  terms in eqs 4 and 7 cancel, eliminating errors that would have been introduced by calculating the gas-phase molecule or radical with BEEF-vdW.

#### 2.2.2 Linear Scaling Relations

The model for the catalytic partial oxidation of methane on Rh(111) from Mazeau et al.^12^ was initially developed with RMG using LSRs to scale the species’ binding energies from Pt(111) data. The thermodynamics on Pt(111) used in the model by Mazeau et al. were also obtained using the BEEF-vdW functional in QE, but the DFT parameters such as plane-wave cutoff energy, *k*-point grids etc., were different from the one used in this work,^53^ and the uncertainties were not reported. To make the LSR-estimated thermodynamic data and uncertainties consistent with the DFT calculations on Rh in this work, the species’ thermodynamic parameters on platinum were recalculated using the same workflow discussed in Section 2.2.1. The polynomial parameters for each species were then modified to describe the thermodynamics based on the LSR correction from Pt to Rh, using eq 8
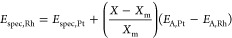
8Where *E*_spec_ is the binding energy of the specific species, and *E*_A_ is the binding energy of the adatom (C, H or O). *X* and *X*_m_ are the bond order and the total possible bond order for the adatom (*X*_C_ = 4, *X*_H_ = 1, *X*_O_ = 2) on Pt(111). The four physisorbed species were not scaled from Pt(111) to Rh(111) because traditional LSRs cannot be applied to those species. Since the binding for each of these species is relatively weak (i.e., *X* = 0), the values and uncertainties were kept the same across all of the models used in this study.

#### 2.2.3 OCP Neural Network Calculator

The relaxed slab and gas-phase molecules were prepared as described in Section 2.2.1. The gas-phase molecules were placed on the Rh(111) and Rh(211) slabs, and the OCP calculator with the GemNet-OC-L-F model^28^ was used in ASE to relax the structures^16^ until the forces were below 0.01 eV/Å. The vibrational analyses were performed in ASE with the same OCP calculator. For the calculation of adsorbate heat of formation at 0 K, The OCP^57^ reported different reference molecules to calculate the heats of formation of gas-phase molecules, so the hypothetical equation was changed to eq 9
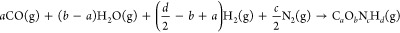
9The workflow described in Section 2.2.1 was adjusted accordingly. To calculate the zero-point-corrected energy of the gas phase reference molecules in eq 9, the atomic gas phase reference energies were from the Supporting Information reported by Chanussot et al.,^57^ and the zero point energies were taken from the experimental values reported in the Computational Chemistry Comparison and Benchmark Database(CCCBDB),^58^ specifically: CO (0.147 eV), H_2_ (0.277 eV), and H_2_O (0.609 eV).

#### 2.2.4 Binding Energy Calculation

As will be mentioned in Section 2.3, the parameters varied in the BPE analysis were the enthalpies of formation of the adsorbates at 298 K. To aid comparison with literature values yet maintain consistency across the models, the prior and posterior distributions reported in the results are converted to binding energies using eq 10.
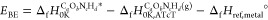
10where  is the heat of formation at 0 K calculated by the aforementioned three methods,  is the heat of formation at 0K of the gas phase counterpart reported in ATcT, Δ_f_*H*_ref,metal_° is 0. The binding energies calculated with DFT values are also detailed in Table S2 of the Supporting Information.

### 2.3 Reactor Simulation

The resulting models with thermodynamics derived from DFT, LSR, and ML were loaded into a simulated packed bed reactor in Cantera.^59^ This reactor was modeled after the experimental setup used by Horn et al.^60^ At the time of our study, Cantera could not solve the differential algebraic equations to simulate a packed bed reactor directly, so a series of 700 continuously stirred tank reactors containing a set catalyst surface area were used to approximate the capillary reactor used in the Horn experiments. The reactor had an inner diameter of 16.5 mm, and a length of 70 mm. The catalyst foam occupied 10 mm of the total reactor length, starting after a 10 mm inlet, and followed by a 50 mm outlet, both containing no catalyst. The porosity of the catalyst was 0.81, and the surface area to volume ratio was 1.6 × 10^–4^ m^2^/m^3^.

The gas feed to the reactor was stoichiometric, meaning the ratio of the molar flow rate of CH_4_ to the molar flow rate of O_2_ was 0.5. Argon was used as an inert carrier gas, with an Ar/O_2_ ratio of 79/21. The total feed flow rate was 4.7 slpm (0.208 mol/min).

The heat transfer for the reactor used by Horn et al. was actually quite complex, as noted in ref (12). Including a fully resolved energy equation for this system would have required assuming a heat transfer model, then iteratively solving the heat transfer equation down the reactor to produce a wall temperature profile, and then resolving this wall temperature with the energy generated within the reactor due to chemical reactions. This iterative approach would have been prohibitively time-consuming for BPE using Monte Carlo methods. Instead, the experimental temperature profile observed by Horn was imposed on the Cantera reactor, and the energy equation was turned off for the simulation.

There are many sources of error in the simulation besides the adsorbate binding energies, such as neglected heat transfer effects, diffusion limitations, coverage effects, and describing the multifaceted catalyst nanoparticles as a pristine (111) or (211) slab. Even a perfect method for predicting the energies is unlikely to perfectly capture the physical system.

### 2.4 Prior and Experimental Uncertainties

#### 2.4.1 Prior Uncertainties

The posterior uncertainties for the surface species in the CPOX model were determined using BPE, which is discussed later in Section 2.5. A wrapper for the Cantera model was constructed that accepted the enthalpies of formation (Δ_f_*H*^C*a*O*b*N*c*Hd^*) for each of the 13 surface species in the model, along with the uncertainties associated with the method used (DFT, LSR, ML) for that particular species. The model outputs were the flow rates for gas-phase CH_4_, CO_2_, CO, O_2_, and H_2_. Only readings over the catalyst bed were taken into account (10 to 20 mm), due to the lack of change in the concentrations over the rest of the reactor.

Because BEEF-vdW is a Bayesian estimation based approach, the uncertainty can be estimated with 2000 perturbations on the exchange–correlation functional.^46^ A Gaussian distribution was constructed with the 2000 energy ensemble, and the 2σ of this distribution was used as the uncertainty for the DFT calculations. The DFT uncertainties were calculated for the gas-phase species used in the hypothetical reaction in eq 3, the slabs, and the adsorbates. The uncertainties were then propagated through eq 7 to calculate the binding energy uncertainty. BEEF-vdW was trained using diverse data sets representing bonding in both chemical and condensed matter systems, so the uncertainty of adsorption systems tends to be overestimated. As such, a factor of 0.683, employed in prior studies,^30,61^ was used to correct the binding energy uncertainty. It should be noted for the DFT uncertainties that the BEEF-vdW functional does not go beyond the generalized gradient approximation for any of the ensemble calculations, and as such is blind to errors and offsets inherent in this functional family. While a truly comprehensive error study would ascend further up Jacob’s ladder, to include other functional families, this was deemed impractical for the current study.

For this study, the uncertainty of the species’ entropy was not taken into account, although it should be noted that this value is not zero.^62−64^ However, for this model, all of the surface species are small molecules with relatively constrained motion across the surface, so it was deemed reasonable to neglect. Thus, only the uncertainty in the species enthalpy was considered. The enthalpic portion of the uncertainty was quantified by determining the uncertainty in each species binding energy, and then using that as the uncertainty in the heat of formation for each species. The uncertainty in the binding energy was supplied as *P*(θ) to the DFT model. The average 2σ error for species calculated using DFT was 0.299 eV.

**Figure 1:**
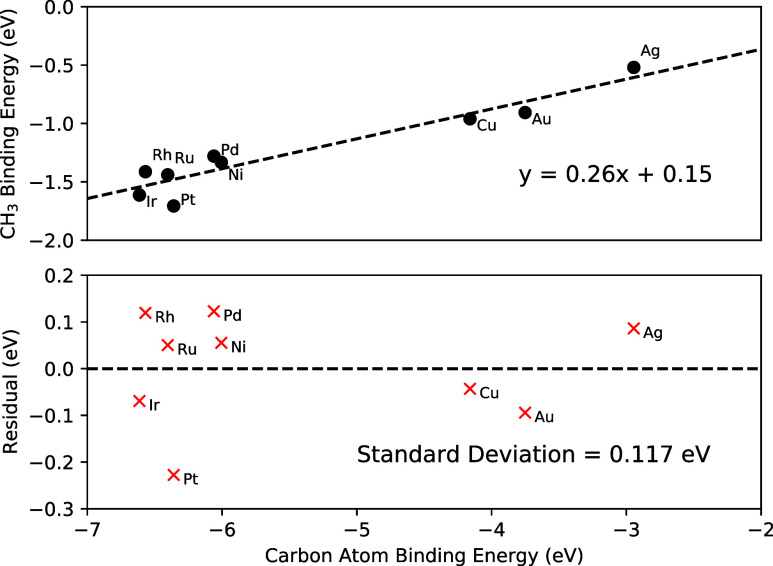
CH_3_ linear scaling relationship plot with data from Abild-Pedersen et al.^13^ (top plot, black circles), along with the residuals for each metal (bottom plot, red crosses) and their standard deviation.

For linear scaling the uncertainties were estimated as follows. There were two sources for the uncertainty: the actual DFT measurements for atomic and species binding energies, and the uncertainty of the linear scaling relations themselves. The values for the uncertainty in the linear scaling trends were derived from the original Abild-Pedersen paper.^13^ This was the sample standard deviation for the trend line residuals for CH, CH_2_, CH_3_, and OH. An example of a linear scaling plot for CH_3_, along with its residuals, is shown in Figure 1.

The aggregate uncertainty was calculated by propagating the error through eq 8 for the species enthalpy
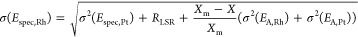
11Where *R*_LSR_ is the residual uncertainty mentioned for the bound atom and bond order corresponding to each species. The average 2σ error for species calculated using the LSR model was 0.424 eV.

The uncertainty for the ML model was estimated using the MAE for the GemNet-OC-L-F model reported in the OC20 data set.^16^ To account for the underlying uncertainty in the original DFT calculations used to train the model, the model MAE (0.239 eV) was added to the DFT error for each species
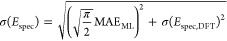
12

**Figure 2:**
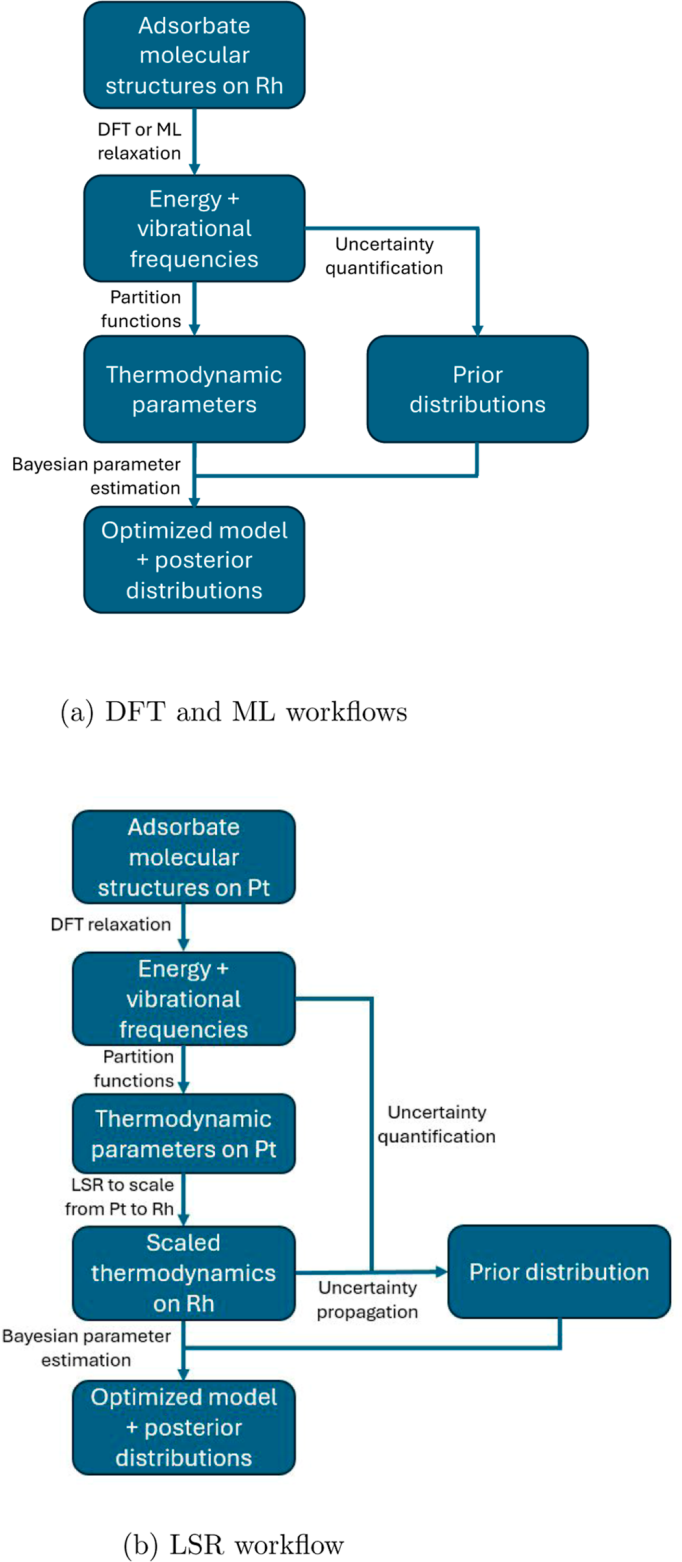
Uncertainty estimation and BPE workflows for (a) DFT and ML models, and (b) LSR models.

The factor of  was included to convert the MAE to a standard deviation, assuming all of the model errors are normally distributed. The average 2σ error for species calculated using the ML model was 0.381 eV. The overall thermodynamic uncertainty workflows for the DFT, LSR, and ML models are shown in Figure 2

#### 2.4.2 Experimental Uncertainties

The error for the output molar flow rates were all taken to be 5% of the total molar flow rate. Horn et al.^60^ report uncertainties in the atom balances for H, C, and O, which gave uncertainties between 1% and 13% for each atom. Unfortunately, there are no details for specific uncertainties at each point in the reactor. Selection of 5% error for all species was somewhat arbitrary, but a convergence study was performed using 10% and 2.5% error, with negligible differences between the models. Details of this study can be found in the Supporting Information

#### 2.4.3 Covariance Matrix Generation

Many parameter errors are correlated in surface chemistry models.^32,65^ To investigate the effect of accounting for the correlation between parameters, this study was conducted using both the uncorrelated 2σ prior uncertainties and a prior covariance matrix. Since BEEF-vdW supplies an ensemble of 2000 values, an estimate of covariance can be obtained by performing the calculations referenced in eq 4 through 7 on each of the energies obtained in the 2000 energy ensemble. This results in 2000 values of Δ_f_*H*_0K,ads_ for each species. Equation 13 was used for each of the 169 pairs of species, resulting in a 13 × 13 grid of values.
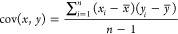
13Where *x* and *y* are the Δ_f_*H*_0K,ads_ entries for each pair of species in the model, and *n* corresponds to the ensemble of 2000 energies generated using the BEEF-vdW functional. The LSR model used a similar method to calculate the covariance matrix, using the ensemble of values generated for each species on platinum instead of rhodium. The residual error from linear scaling (*R*_LSR_) in eq 11 was not used as a single value in the uncertainty calculations. Instead, each member of the 2000 member ensemble was run through the linear scaling eq 2 million times, using a random sample from a normal distribution with σ = *R*_LSR_ in place of the single value used for the residual error. The resulting ensembles of binding energies for each species were then run through eq 13 to generate the covariance matrix.

### 2.5 Parameter Estimation and Uncertainty Quantification

BPE requires prior knowledge of uncertainties for both the model inputs and outputs, as can be seen in Bayes theorem (eq 14)
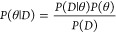
14In this case θ represents a vector of the surface species enthalpies of formation (Δ_f_*H*^C*_a_*O*_b_*N*_c_*H_d_^*) and *D* is the observed output flow rates for CH_4_, CO_2_, CO, O_2_, and H_2_. *P*(θ), or the prior, represents the probability distribution of the species enthalpies of formation from Section 2.4.1 and *P*(*D*) represents the probability distribution of the experimental mole fractions determined in Section 2.4.2. The likelihood *P*(*D*|θ) and the posterior *P*(θ|*D*) are both distributions themselves, and can be generated by creating successive samples of the prior values and running them through the simulated reactor specified in Section 2.3. Sampling of the posterior was performed using MCMC, which proposes random jumps in parameter space and either proceeds with the jump or does not based on the probability of the jump destination. This algorithm was used to sample the HPD region of the posterior. MCMC sampling was performed using the Zeus package,^39^ which used a modification of the MCMC algorithm known as ensemble slice sampling.^66^ This approach allowed MCMC chains to run in parallel, and communicate information about all active chains (i.e., the ensemble) to determine the direction of the next jump in parameter space. The Zeus package, as well as utilities for inputting priors, experimental data, and other parameters necessary for BPE, are conveniently wrapped in the Parameter Estimation and Uncertainty Quantification for Science and Engineering (PEUQSE) package,^67^ which was employed for this study. 52 independent MCMC chains ran in parallel on 52 CPU cores, with an initial distribution spread of 0.25 and a filter coefficient of 1.0.

For a well sampled system, the maximum of the a posteriori distribution (MAP), i.e. the most likely values for a given parameter set, should fall within the HPD region. For BPE, the MAP values and their corresponding HPD regions represent the “feasible set” of values and their uncertainties when both the experimental data and prior uncertainties are considered. The quality of sampling was quantified by examining the autocorrelation time (ACT) for each chain, along with observations that the HPD region and MAP were converged at stable values.

Most of the posterior distributions were deemed to be well-sampled within ∼100,000 samples post burn in. The exception was the correlated DFT model, which required ∼1,000,000 samples before the ACT assumed a stable value for all chains. The ACT plots for each model are in the Supporting Information.

## 3 Results and Discussion

### 3.1 Thermodynamic Data

There are 13 adsorbates in the model, none of which are larger than C1. As mentioned in Section 2.4.1, small molecules adsorbed on a surface generally have negligible contributions from their rotational and translational modes, so the uncertainty in the entropy was not considered. Consequently, only the uncertainties of species enthalpy were estimated and considered in the reactor simulations. All the thermodynamic data estimated by the DFT, LSR and ML models for each species can be found in the Supporting Information.

**Figure 3:**
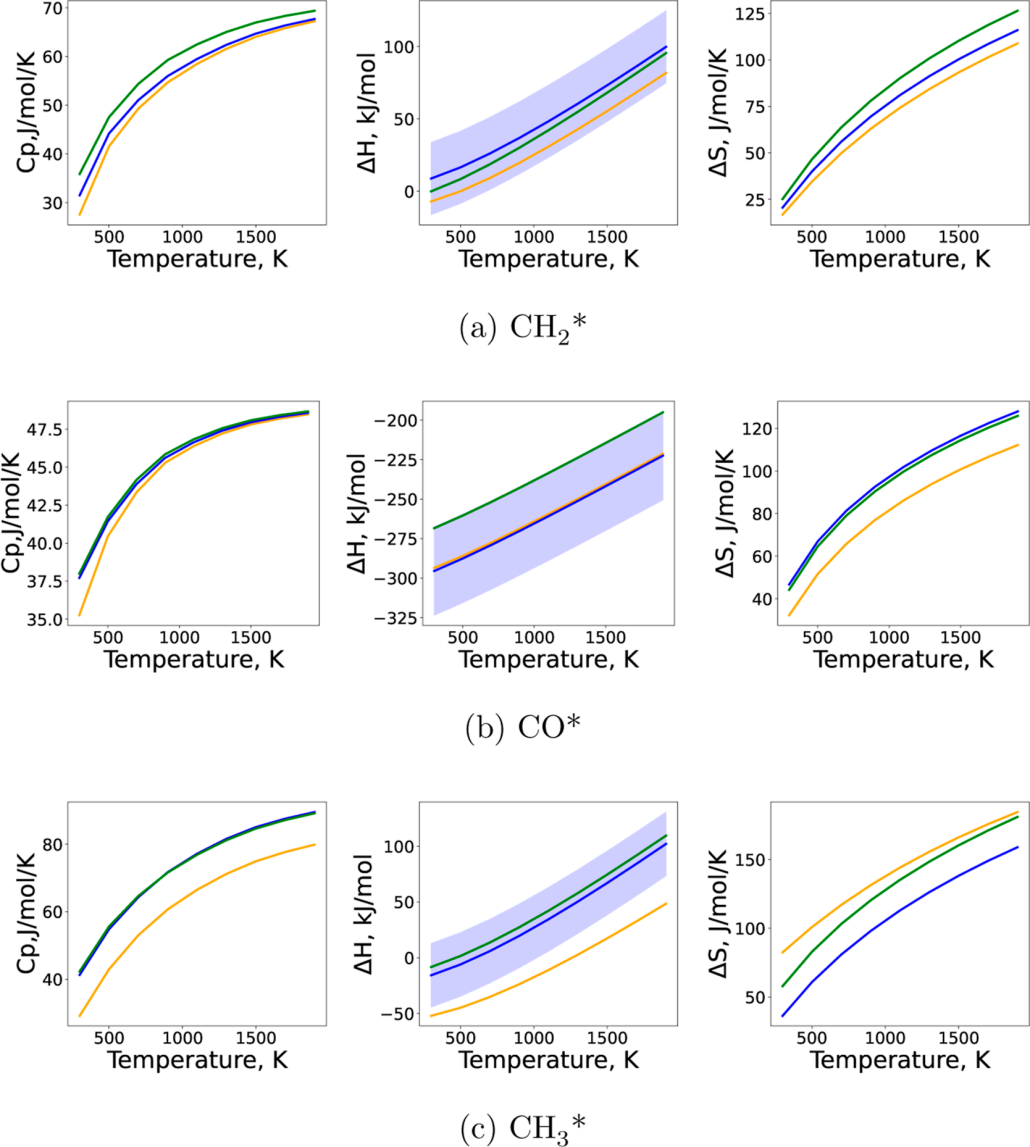
Heat capacity, enthalpy, and entropy comparison for three species estimated through DFT (blue), LSR (orange), and ML (green), the blue band represents the uncertainty of the DFT method.

Similar to the enthalpy for adsorbed CH_2_ in Figure 3a, the enthalpy obtained through DFT, LSR, and ML are very close to the DFT uncertainty for most of the species in the model. All the ML estimated enthalpies are within the uncertainty of the DFT data. However, the enthalpy of CH_3_* estimated by LSR is outside of the DFT uncertainties as shown in 3c. The largest enthalpy of formation difference between ML and DFT is 27 kJ/mol for CO* (Figure 3b), while largest enthalpy disagreement between LSR and DFT is 34 kJ/mol for CH_3_* (Figure 3c). The thermodynamic comparison for the rest of the species in the model can be found in the Supporting Information.

**Figure 4:**
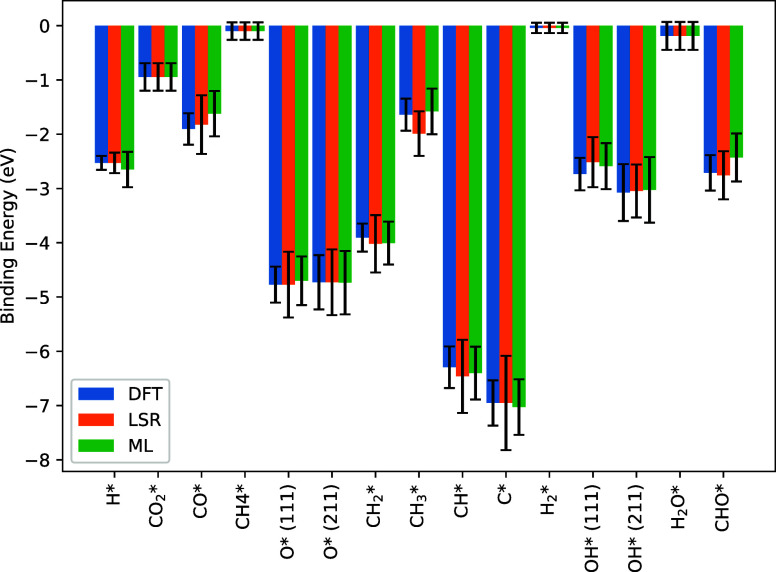
Binding energy and uncertainty of each species estimated by DFT, LSR and ML methods. The 2σ values of the prior distribution are used as the error bars in black.

Figure 4 and Table 1 compare the binding energy and the uncertainty of each species estimated by the three methods. For the physisorbed species (CO_2_*, CH_4_*, H_2_*, and H_2_O*) the DFT values are used for all three models. The convention recommended by Ruscic^68^ for thermochemical uncertainties is used in Figure 4 and throughout this paper, i.e. reporting the 2σ values as the uncertainties, since they encompass approximately 95% of a normal distribution. These uncertainties were used as the prior distribution in the BPE, as described in Section 2.5. The largest binding energy disagreement estimated by LSR and ML compared to DFT is about 30 kJ/mol among all the species in the model, so using the LSR and ML models to generate the thermodynamic data in microkinetic modeling is reasonably reliable compared to DFT results for small molecules, considering the relative uncertainties inherent in DFT.

### 3.2 Optimized Thermodynamics

**Figure 5:**
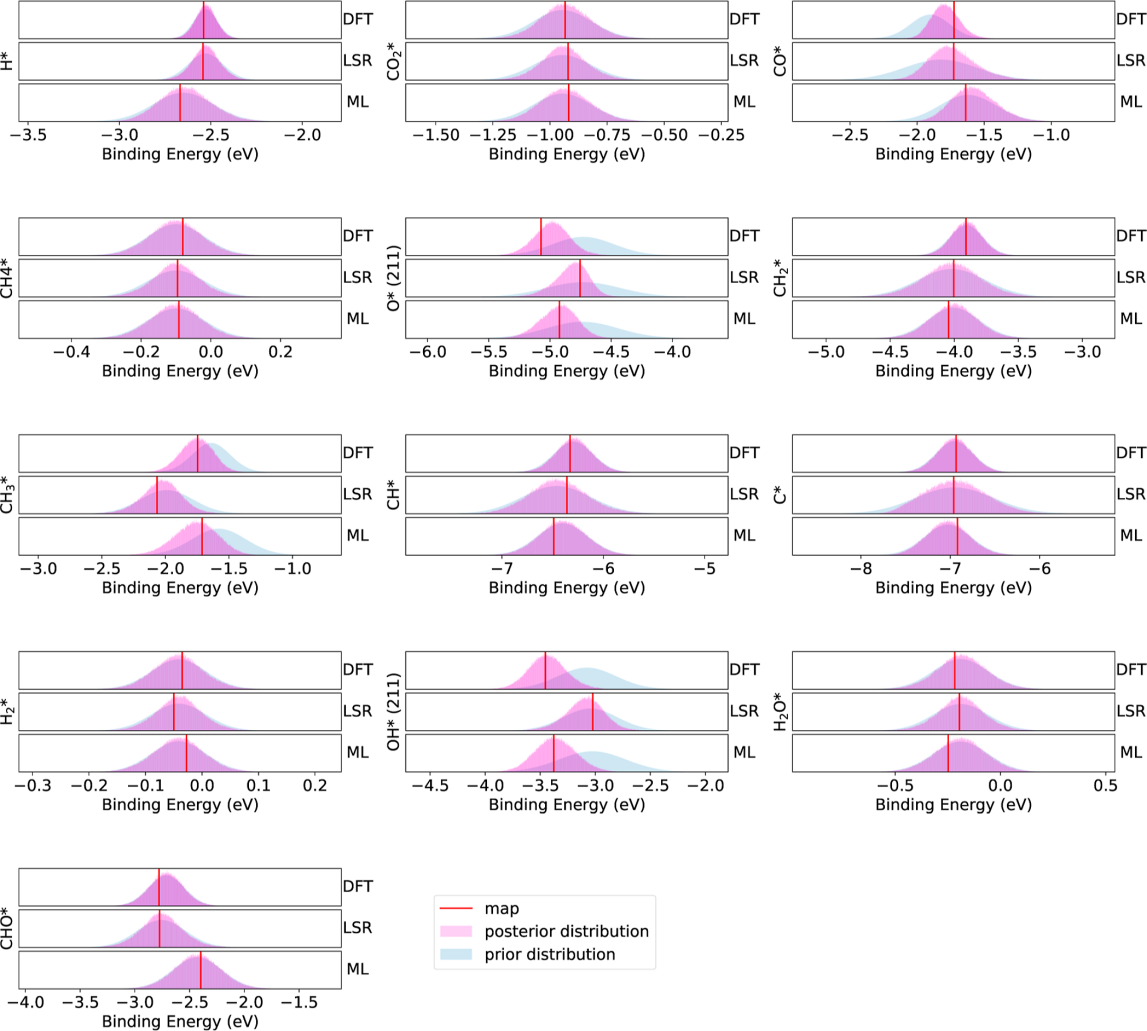
Prior distribution (blue shaded area), posterior distribution (pink shaded area) and the MAP value (red vertical line) for every adsorbed species in the model.

The prior and posterior probability densities for each of the species used in the model can be found in Figure 5, along with the MAP values for the species binding energies.

**Figure 6:**
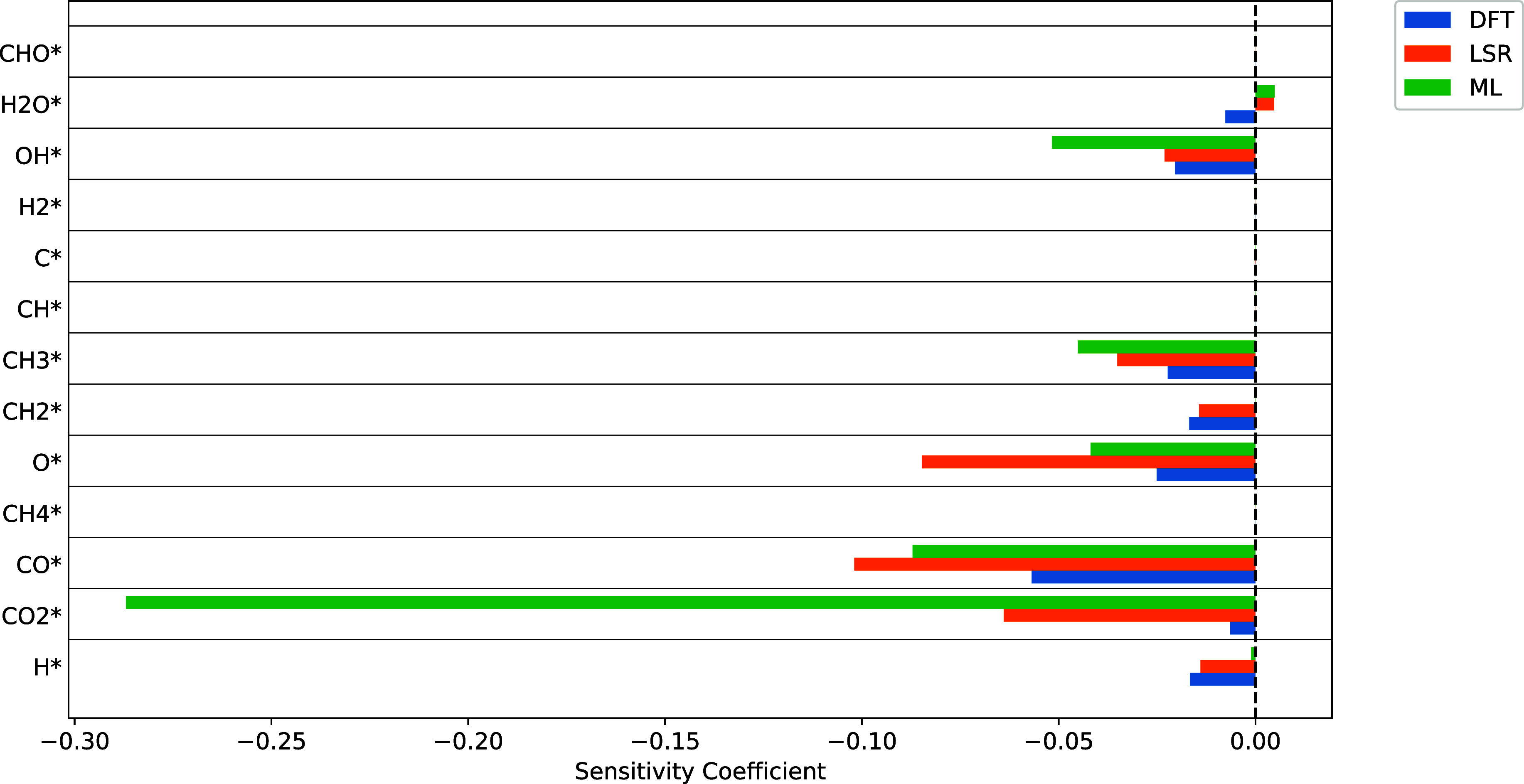
Sensitivity plot of CH_4_ conversion to the binding energy of each species.

On the whole, the posteriors for all of the species are symmetric, with the exception of OH*, O*, CO and CH_3_. It is quite surprising that these species are the only ones with asymmetric posteriors, which are quite common in similar studies^35,36,69^ due to the complex and nonlinear dependence of reaction rates on the species enthalpies. For some species, the lack of skewness and kurtosis in the posterior can be explained by examining the sensitivities of the species within the model. In general, physisorbed hydrogen (H_2_*) and methane (CH_4_*), along with adsorbed carbon (C*), methylidine (CH*), and formyl (CHO*) have little effect on the outlet molar flow rates or model observables. Figure 6 shows the first order sensitivities of the methane conversion to all of the species in the model. The sensitivity plots for a number of other observables (CO/CO_2_ selectivity, O_2_ conversion, etc.) can be found in the Supporting Information, but overall a lack of sensitivity to these species was observed for all benchmarks used. Since the model is insensitive to these species, their posterior distributions are very similar to their prior distributions.

There is a trend in the CH_3_* posteriors to have a slightly stronger binding energy than predicted a priori for all three models. Prior studies with γ-Al_2_O_3_ supported rhodium nanoparticles show evidence that (1) the active sites are on the metal surface for this mechanism, not the support,^70,71^ and (2) CH_4_ conversion both with and without gas-phase O_2_ increases with decreasing particle size, which indicates that the active sites are likely edge/step sites. The lower coordination of the step sites means that species will likely bind more tightly to them.

The opposite trend is observed for CO*, with the posteriors showing a slightly weaker binding energy than the priors. It is possible that this is due to coverage dependence not being included in the microkinetic model. Higher oxygen coverage at earlier times in the reactor would limit the rate of formation of CO and the adsorption of CH_4_. Since the model was optimized only with respect to the species binding energies, the CO binding energy may have been weakened to account for this. Nevertheless, the differences in both the CO and CH_3_ species was slight for the DFT model, indicating that the initial DFT calculations were at least close to the values predicted by the model.

**Figure 7:**
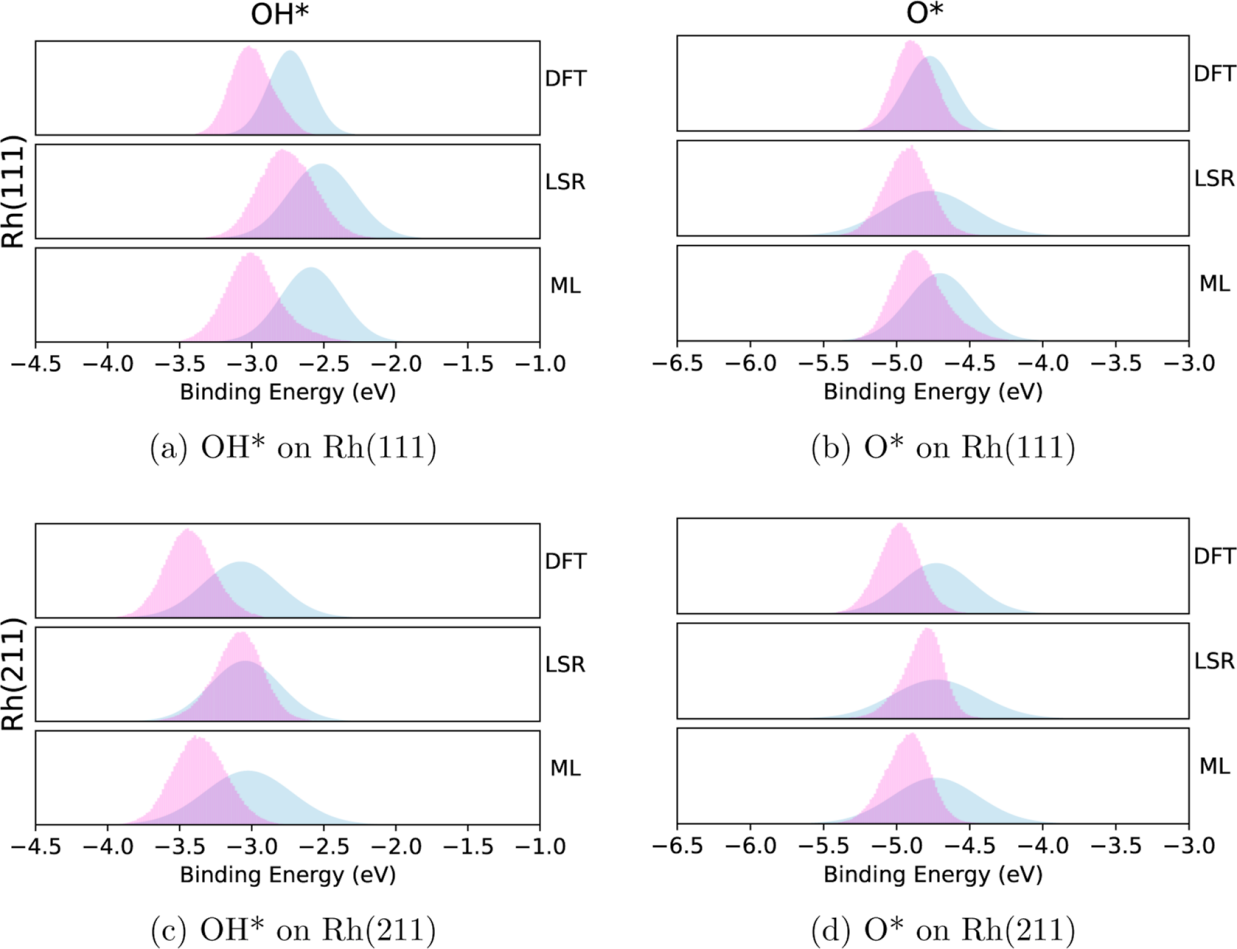
Comparisons of the prior and posterior distributions for OH* (left) and O* (right) on Rh(111) (top) and Rh(211) (bottom) sites. The prior distributions are shown in blue, the posterior distributions are shown in red.

The posterior distributions of OH* and O* for the initial model using Rh(111) showed much stronger binding energies than the priors supplied. Given that OH* binds stronger (more negative binding energy) on the (211) facet than (111) as reported by Yang et al.,^72^ the possibility of these species having an alternative binding site was considered. As such, the DFT, LSR, and ML models were updated with calculations for these species on their corresponding lowest energy binding site on Rh(211). Figure 7 shows a comparison of the priors and posteriors using both the Rh(111) and Rh(211) binding energies for these species.

The calculations for OH* show a dramatic shift in the posterior when the (211) site is considered, with the MAP value changing by approximately 0.4 eV between the (111) and (211) posteriors in the DFT model. The shift for O* was less dramatic, changing by only 0.11 eV. Literature values show a less dramatic shift as well for oxygen,^13,73^ which is likely due to its hollow binding site on both (211) and 111, versus OH which prefers the (211) step edge.^51^ The binding energy values optimized through BPE closely align with those reported for the (211) site on rhodium by Abild-Pedersen et al., although given the priors, it could be said that the actual lowest energy site may have an even stronger binding energy, such as a defect site. Further study is required to validate this claim, but the simple result of this work shows that both O* and OH* prefer a lower energy site than initially predicted in the (111) model, which is a useful result.

It should be noted that the prior and posterior values for the binding energies shown in this paper have been calculated using the method described in Section 2.2.4, which uses the enthalpy of formation at 0 K from the ATCT for the gas phase precursor. This was done so that the priors and posteriors for all three methods could be compared using the same gas phase reference, but this method does mean the binding energies reported will be offset from values reported from other sources. Typically, DFT-calculated electronic energies are used in eq 10. Using the latter method, the DFT binding energy for O* on Rh(111) is −4.96 eV and on Rh(211) it is −4.90 eV. This is quite close to reported literature values of −4.87^74^ and −4.90 eV.^13^ Similarly, the calculated binding energies for OH* on Rh(111) and Rh(211) are −2.87 and −3.22 eV respectively, with literature values of −2.87^74^ and −3.26 eV.^13^ All species binding energies calculated using the DFT calculated electronic energy can be found in the Supporting Information.

It is noteworthy that the (BPE) performed using the Rh(211) DFT, LSR, and ML model values show very similar posteriors, despite the differences in their priors. This convergence signifies a high level of confidence in the optimized value.

### 3.3 Simulated Responses

**Figure 8:**
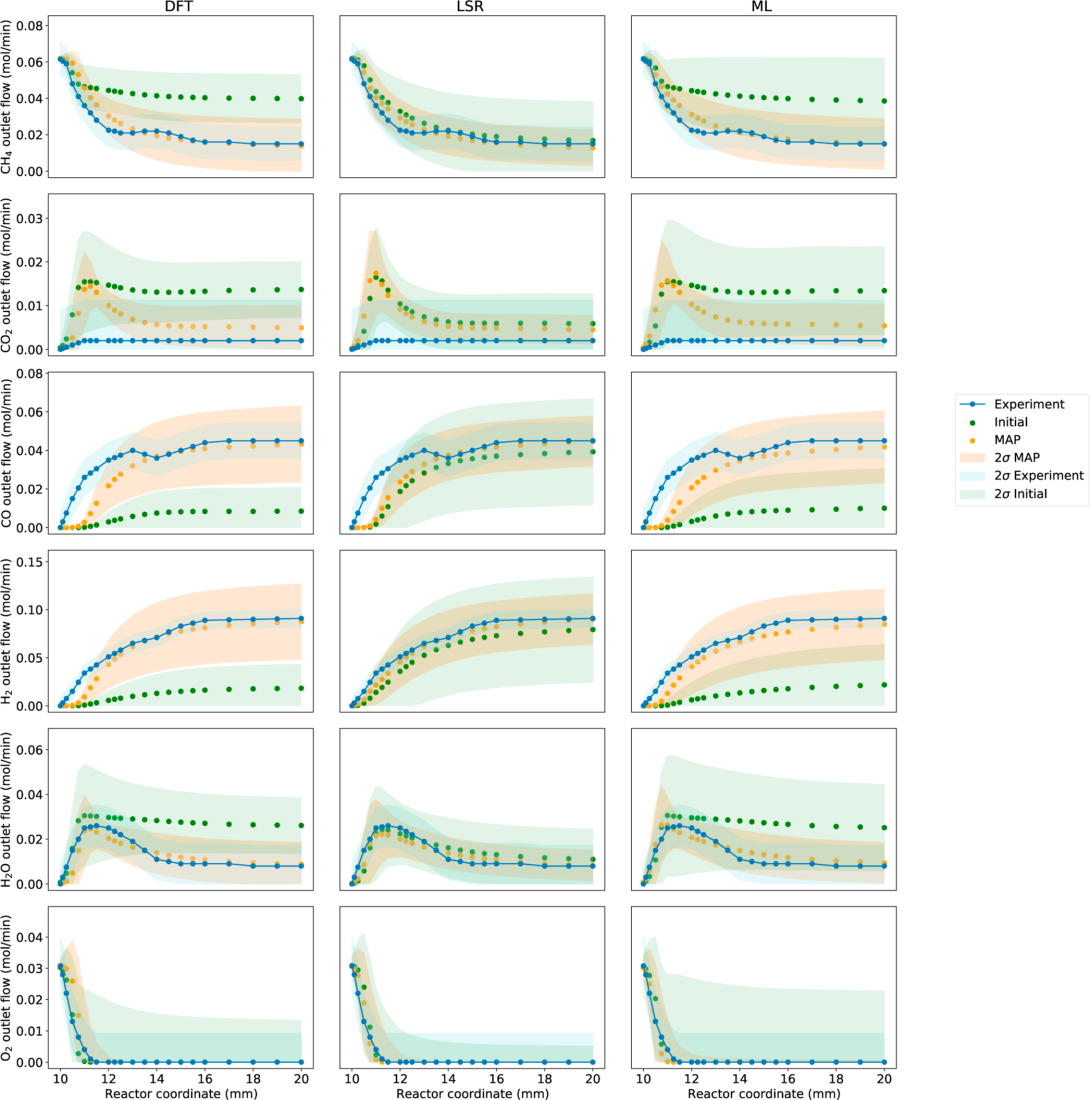
Gas phase flow rates observed in the unoptimized model (green marker), the optimized model (orange marker), and the experimental data reported by Horn et al.^60^ The shaded regions are the 2σ (95% confidence) intervals for the uncertainty in the experimental data (light blue region), the MAP model (light orange region) and the initial model prior to BPE (light green region).

The sampling of the HPD was used to generate the posterior distribution for the gas phase species profiles within the catalyst bed, as seen in Figure 8. All three thermodynamic estimation methods converged on very similar profiles, with the main difference between them being the posterior uncertainties.

The initial model generated using LSRs is quite close to the experimental profiles compared to the DFT and ML models. This may be due to how the original model from Mazeau et al.^12^ was constructed. RMG uses linear scaling relations, which may have led to selecting rate rules, reaction trees, and thermodynamic trees that generate more accurate results with thermodynamics generated from linear scaling relations.

The initial DFT and ML models are both quite similar, which is likely due to the closeness of their prior parameter values. The largest difference between the two is the magnitude of their uncertainties, which may be overestimated for the ML model, as stated in Section 2.4.1. Nevertheless, it is remarkable how close the solutions for the DFT and the ML model are.

The pathways of three optimized models can be found in the Supporting Information. All the pathways were the same with almost identical conversion rates. This further confirmed that the optimized models converged on similar mechanisms.

### 3.4 Covariance

All of the runs mentioned in the preceding sections used uncorrelated uncertainties for all of the mechanisms analyzed. This is not reflective of the true system, especially for the case of linear scaling relations, where species share a very clear linear correlation with other species bound through the same atom. The covariance matrices for the DFT and LSR mechanisms were constructed using the BEEF-vdW ensembles mentioned previously. As described in Section 2.4.3, each of the equations in Section 2.2.1 were applied for all 2000 rows of the ensemble calculations, and then the covariance was calculated for each species from the resulting 2000 heats of formation. The resulting prior and posterior covariance matrices can be found in the Supporting Information. The model could not be supplied with a covariance matrix.

For the LSR model, the posteriors were all very similar to the uncorrelated case, with MAP values all falling within ±0.1 eV for each model parameter. The DFT model showed more significant deviations when correlated priors were used. Detailed contour plots for all of the DFT and LSR models are in the Supporting Information. The differences between the prior and posterior were quantified using the Kullback–Leibler (KL) divergence test.^75^ Comparing the relative KL divergences gives a quantitative measure of the information gained from the experimental data.

**Figure 9:**
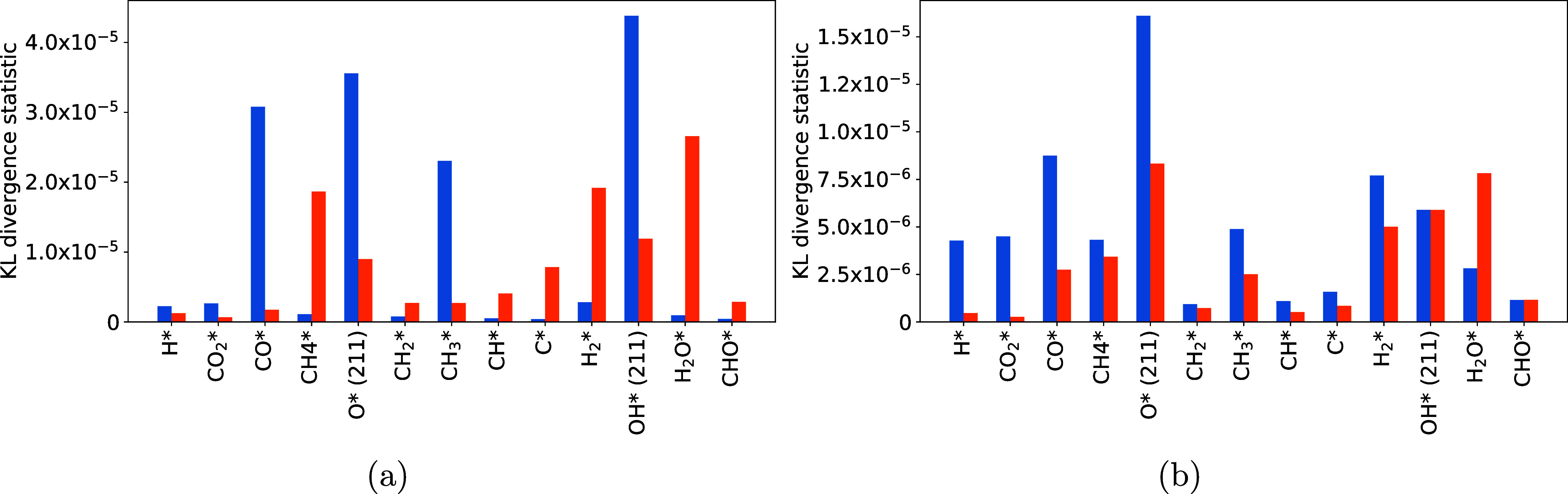
Comparison between the KL divergence statistics for (a) the DFT models and (b) the LSR models. Blue bars are the KL divergence statistic for the models with no covariance in the prior. Orange are the statistics for the models including covariance.

**Figure 10:**
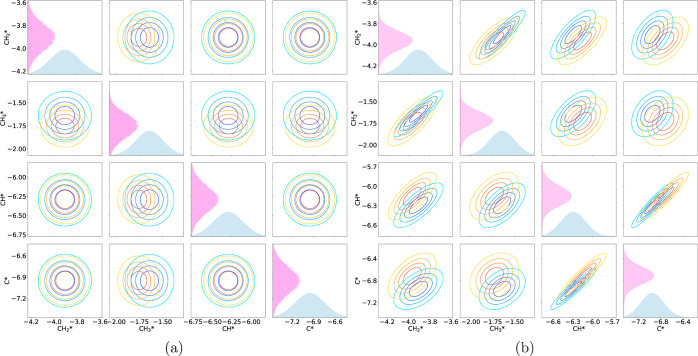
Contour plots for carbon bound species in the DFT models (a) without covariance and (b) with covariance. On the diagonal are the prior distribution (blue) and the posterior distribution (pink). Decreasing contours show a 20% decrease in probability.

**Figure 11:**
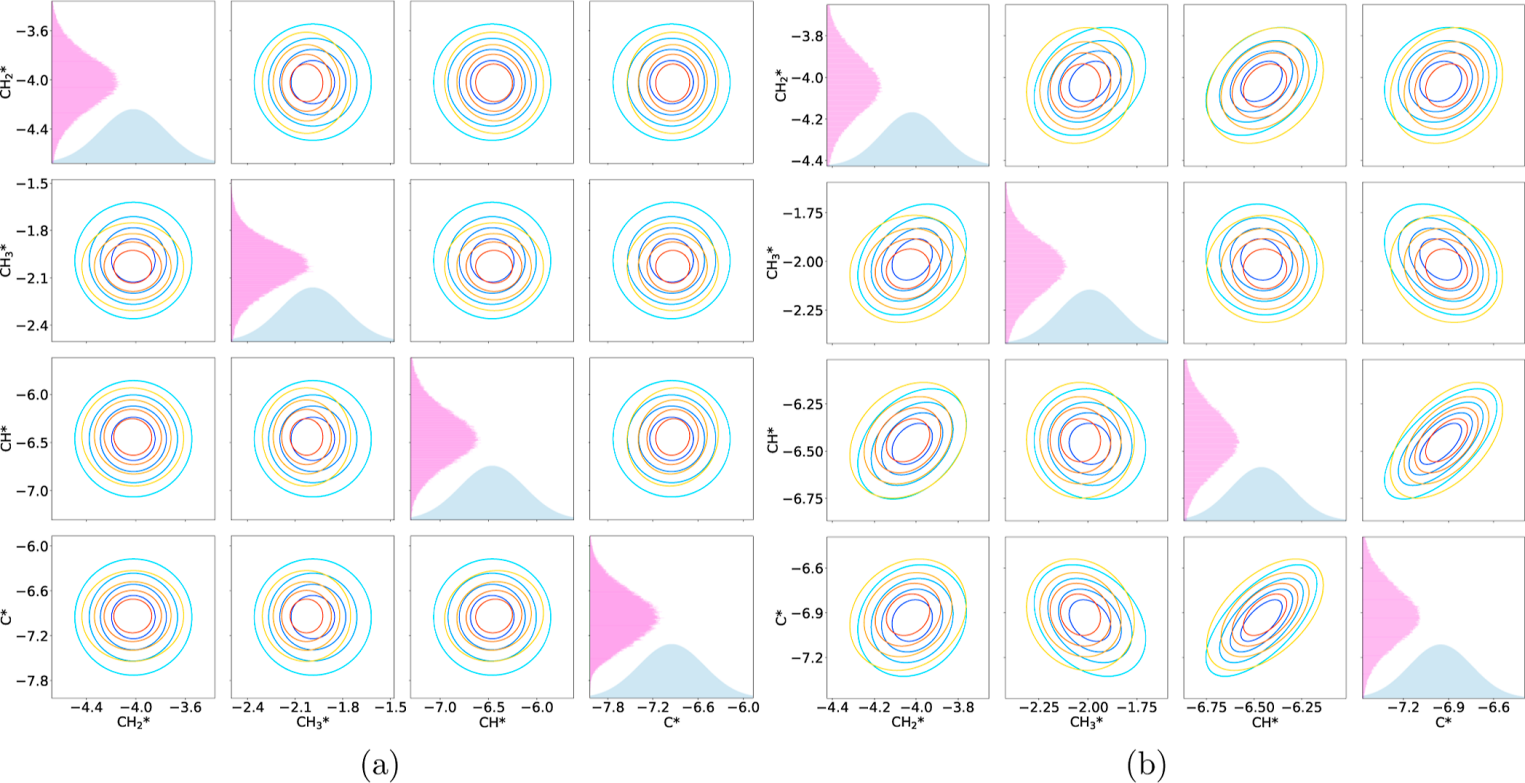
Contour plots for carbon bound species in the LSR models (a) without covariance and (b) with covariance. On the diagonal are the prior distribution (blue) and the posterior distribution (pink). Decreasing contours show a 20% decrease in probability.

Figure 9 shows a comparison between the information gained from using covariance for the DFT model (Figure 9a) and the LSR model (Figure 9b). There are two clear trends that can be seen. First, between both the DFT and the LSR model, when covariance is considered, the KL divergence statistic decreases. Since we are only using the 1D probability distributions to calculate the KL divergence statistic, this is expected. Including covariance constrains the parameter posteriors. This is illustrated in Figures 10 and 11.

While it is more drastic in the DFT case, it is clear that the inclusion of covariance restricts where the posterior can be located. This reduces the search space, which means that overall there is less information to be gained from the experimental data to inform the posterior.

The second trend between the two is best shown when examining the DFT model. Some species overall have a higher KL divergence statistic when covariance is considered. CH_4_*, CH_2_*, CH*, C*, H_2_*, H_2_O* and CHO* all show a larger degree of information gain. The physisorbed species are all correlated inherently in the BEEF-vdW functional, and the species bound through carbon have strong correlations with each other. Thus, even if certain species gain less information on their own because they are not as consequential to the model, they can gain information from other more important species that they are strongly correlated with. For the physisorbed species, this key species is likely CO_2_. For species bound through carbon, this may be CH_3_ and CH_2_. This trend is not seen in the LSR model, where only H_2_O exhibits a larger KL divergence statistic when covariance is considered. The LSR prior parameters generated simulation data that are incredibly close to the optimized model, so it is likely that including covariance had little effect in general.

The remainder of the posterior distributions for the models including covariance can be found in the Supporting Information. Overall, the inclusion of covariance did not change the conclusions about the LSR and DFT models concerning the alternative binding sites for O* and OH*. Indeed, the posterior distributions for O* and OH* had slightly stronger MAP binding energies using correlated priors than was predicted by the uncorrelated models. Supplying a covariance matrix for the OCP data, if it were possible, would likely not have changed the answers significantly.

### 3.5 Conclusions

The first goal of this study was to analyze the uncertainty inherent in different thermodynamic estimation methods for adsorbed species. It is clear that the ML model is on par with the LSR and DFT models, with the additional benefit that the thermodynamics did not require any expensive DFT calculations up front, which cannot be said for the other two models. Knowing the uncertainty of the predictions from a calculator before using it to screen thousands of catalysts is vital for any method. Given the large shifts from the prior values to the MAP values for all 3 models, the apparent increase in uncertainty for the ML model is made up for by its speed compared to DFT and universality compared to LSRs. Further, for the system at hand, we showed that while supplying covariance data was useful for having slightly more accurate posteriors, it did not significantly change the final MAP values or the optimized models. This is encouraging, as the data necessary to determine covariance are not always available, especially when dealing with mechanisms that use data from a variety of sources, for example those generated by the RMG.

The secondary goal was to validate that BPE used in conjunction with tools like the OCP calculator could be useful for catalyst screening. The finding that OH and O likely have stronger binding energies than the values reported on Rh(111) shows how informed optimization can improve a microkinetic model that was initially constructed from chemical intuition or automated construction methods. Coupling uncertainties calculated using BPE with rapid screening methods would allow for a more informed exploration of complex chemical spaces, as opposed to using the baseline values from surrogate models like linear scaling relations and machine learning models.

Finally, It should be noted that Monte Carlo methods are a taxing way to optimize a model and quantify error. While this study does show the computational efficiency of using a ML calculator to replace DFT, exploring the uncertainty space of the resulting model was costly. Specifically, the chain length required for convergence on each model (approximately one million points) took 52 CPU cores approximately 3 days of computing time. In light of this, it would be impractical to run BPE for every point within a screening study. It is more important to apply it to experimental data that are well-known, and then using the error bars obtained to extrapolate to different systems.

The exploration of the HPD region would be required regardless of the thermodynamic calculation method used, so it is worth mentioning how well each method compares when examining the raw time needed to get the initial estimates. The cost of the DFT calculations on Rhodium was about 1500 GPU hours and 10,600 CPU hours, and the cost of the DFT calculations on Platinum for LSR fitting was about the 1200 GPU hours and 8000 CPU hours. In comparison, the expense of the ML predictions can be essentially neglected, as they can give a structure and energy in minutes with minimal resources. Considering the convergence of BPE to a similar optimized model across all three estimation methods, using ML-aided estimation is considerably more efficient. While some species will require DFT calibration, initiating from ML estimates serves as a valuable starting point, offering substantial time and computational resource savings.

## Data Availability

Associated scripts can be found on the Github repository https://github.com/comocheng/cpox_uncertainty

## Supporting Information Available

Mechanism barrier information for select reactions; integrated flux diagrams for the microkinetic models used; prior, posterior and simulated output plots for 10% experimental error and 2.5% experimental error; prior, posterior and simulated output plots for all species on Rhodium 111; prior and posterior covariance matrices; prior and posterior covariance contour plots; autocorrelation time plots; thermodynamic sensitivity of CO selectivity, CO yield, full oxidation selectivity, full oxidation yield, H_2_ selectivity, H_2_ yield, CH_4_ conversion, O_2_ conversion, syngas selectivity, and syngas yield (PDF)
Cantera YAML input files for the MAP mechanisms and xyz files for the relaxed dft structures (ZIP)
