# Supplementary material for: Uncertainty Quantification of Linear Scaling, Machine Learning, and Density Functional Theory Derived Thermodynamics for the Catalytic Partial Oxidation of Methane on Rhodium
Source: J Phys Chem C Nanomater Interfaces. 2024 Oct 3;128(41):17418–33. doi: 10.1021/acs.jpcc.4c05107 (PMC11492380; doi:10.1021/acs.jpcc.4c05107)
Supplement: Supplementary file 2 — jp4c05107_si_002.zip [file jp4c05107_si_002.zip › supplementary materials/supplementary_material.pdf]

Supporting Information

Uncertainty Quantification of Linear Scaling, Machine Learning, and DFT Derived Thermodynamics for the Catalytic Partial Oxidation of Methane on Rhodium

Christopher J. Blais, Chao Xu, Richard H. West

Department of Chemical Engineering, Northeastern University, Boston, MA, USA

Email address: r.west@northeastern.edu (Richard H. West)

Table S1: Comparison of the DFT, LSR, and ML mechanism barriers vs literature. units are in kJ/mol

Reaction	Filot (2015)	Kraus (2017)	Hickman (1993)	$E_{a,DFT}$ (400K,1200K)	$E_{a,LSR}$ (400K,1200K)	$E_{a,ML}$ (400K,1200K)
CH4 + 2 X $\Longleftrightarrow$ CH3X + HX	36.0	20.0	N/A	49.9,82.6	68.9,112.5	47.2,79.2
CH2X + HX $\Longleftrightarrow$ CH3X + 2 X	33.0	57.0	N/A	0.0,0.0	0.8,0.0	0.0,0.0
HX + CHX $\Longleftrightarrow$ CH2X + 2 X	78.0	112.0	N/A	59.6,53.8	61.0,56.4	73.0,67.2
CHX + X $\Longleftrightarrow$ HX + CX	91.0	0.0	N/A	26.7,31.2	31.2,34.9	55.9,60.5
CX + OX $\Longleftrightarrow$ COX + 2 X	92.0	60.0	N/A	2.7,2.5	4.1,3.8	1.8,1.7
COX + OX $\Longleftrightarrow$ CO2X + X	80.0	79.0	105	120.2,114.9	124.0,118.3	135.1,129.4
CHOX + 2 X $\Longleftrightarrow$ HX + COX	17.0	0.0	N/A	0.7,1.3	1.4,1.9	0.0,0.2
OX + H2OX $\Longleftrightarrow$ OHX + OHX	53.0	155.0	N/A	17.1,19.2	4.3,5.5	4.0,5.0
HX + OX $\Longleftrightarrow$ OHX + X	156.0	185.0	84	1.5,0.2	0.0,0.0	0.0,0.0
HX + OHX $\Longleftrightarrow$ H2OX + X	108.0	60.0	34	87.6,81.6	104.6,98.2	106.7,100.0

0.1. Activation Energy Comparison

Table S1 shows a comparison between the activation energies in the MAP models for DFT, LSR, and ML, along with the same barriers used in published mechanisms by Filot [S1], Kraus [S6], and Hickman [S3].

0.2. Integrated Mass Flux

Below in Figure S2 are the integrated mass flux diagrams for species fluxes across the entire PFR.

0.3. Results of Analysis with Rh(111)

As mentioned in the paper, the initial mechanism tested used the thermodynamic parameters for O and OH adsorbed on 111. The full results for the species priors and posteriors (Figure S3) and the model output molar flow rates (Figure S4 can be found below.

0.4. Experimental Error Validation

The experimental data reported by Horn et al. [S4] had an assumed uncertainty of ±5%. The impact of this assumption was tested by assuming a low range uncertainty of ±2.5% and a high range uncertainty of ±10%. The results of these runs are shown below in Figures S5 through S8. The changes between the ±2.5%, ±5%, and ±10% were minimal.

0.5. Covariance analysis

Below are the prior (Figure S9 and posterior (Figure S10 covariance matrices for the the DFT, LSR, and ML models. It should be noted that the ML model did not include covariance. Additionally, contour plots are included for the DFT and LSR models (Figures S11 and S12, as well as the output prior posterior comparison (Figure S13) and the model output mole fractions (Figure S14

0.6. BPE Convergence

Convergence for MCMC simulations can often be judged by how close the MAP value is to the mean of the posterior distribution (μ_{AP}). In this particular case, the effect that changing any species binding energy has on the overall microkinetic model is complex, and in this case resulted in several asymmetric posteriors. PEUQSE has several integrated convergence diagnostics that were used instead, namely Autocorrelation time (ACT). Autocorrelation time was analyzed using the zeus package [S5], and generally, it can be interpreted as the number of samples required before the starting point of the chain is no longer relevant [S2]. This can visually be interpreted from a plot of the number of samples vs the estimated τ, where a converged MCMC chain will approach an optimal value of τ after enough samples have been performed. The autocorrelation times corresponding to each species in the model can be seen in Figure S15

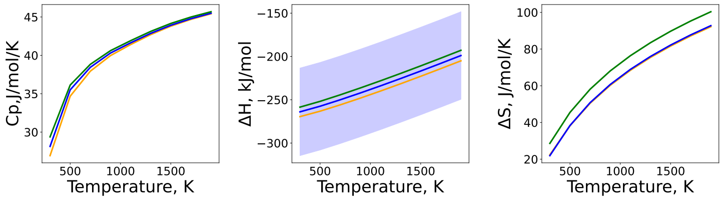

(a) OH*

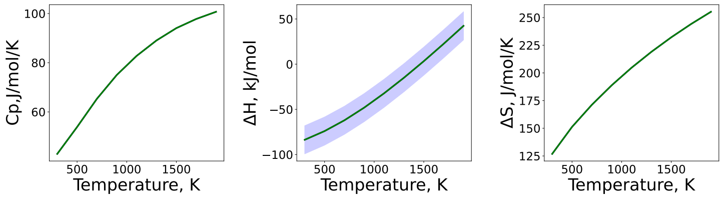

(b) CH_4*

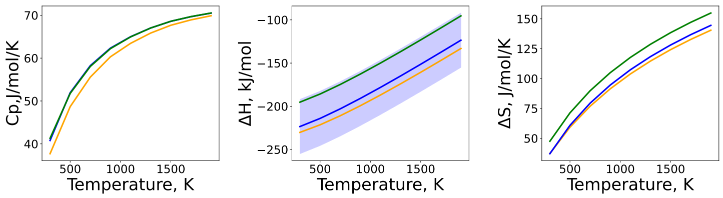

(c) CHO*

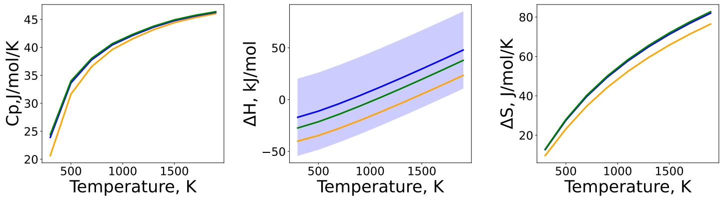

(d) CH*

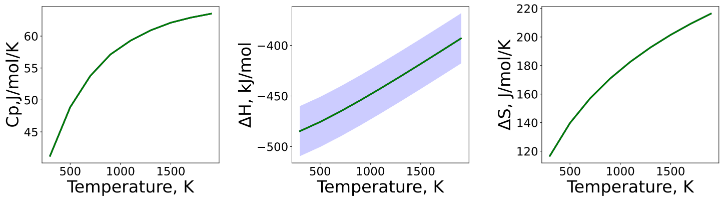

(e) CO_2*

Figure S1: eat capacity, enthalpy, and entropy comparison for three species estimated through DFT (blue), LSR (orange), and ML (green), the blue band represents the uncertainty of the DFT method, VDW species are only

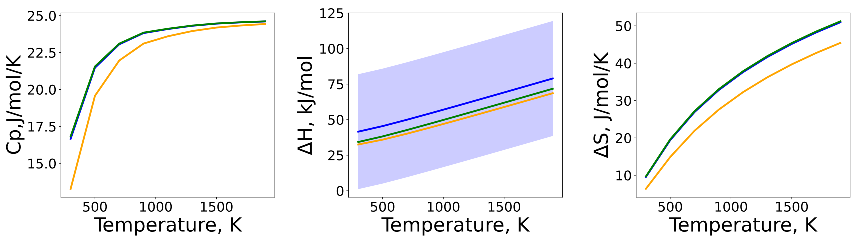

(f) C*

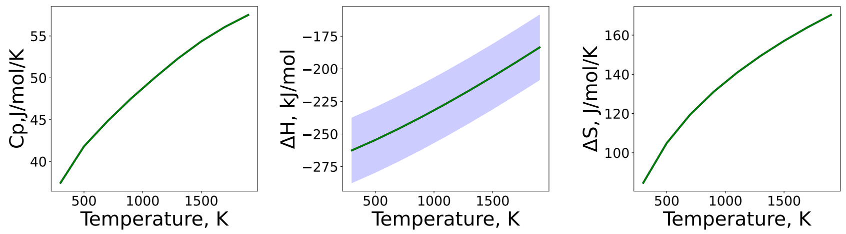

(g) H_2O*

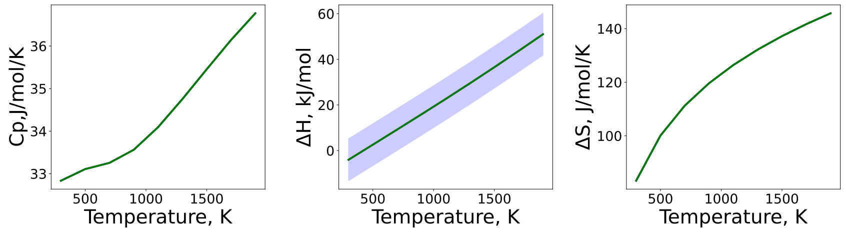

(h) H_2*

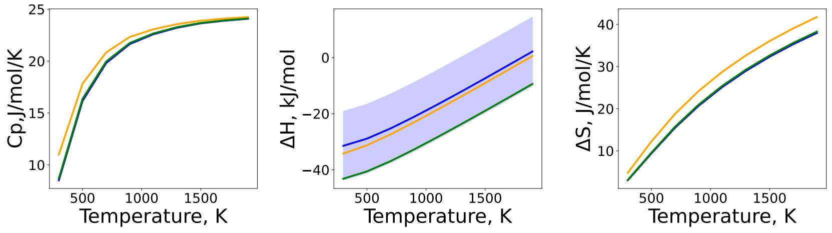

(i) H*

Figure S1: Heat capacity, enthalpy, and entropy comparison for three species estimated through DFT (blue), LSR (orange), and ML (green), the blue band represents the uncertainty of the DFT method

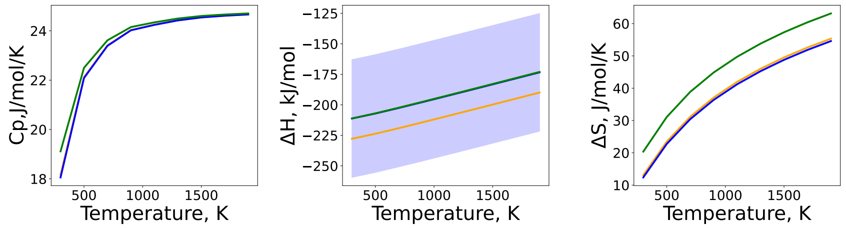

(j) O*

Figure S1: Heat capacity, enthalpy, and entropy comparison for three species estimated through DFT (blue), LSR (orange), and ML (green), the blue band represents the uncertainty of the DFT method

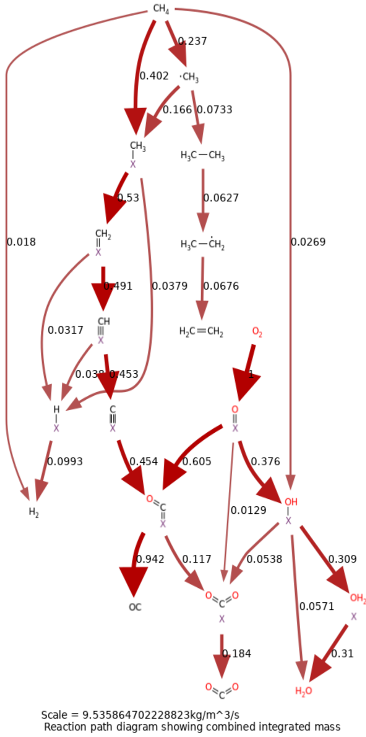

(a) Optimized DFT model reaction path

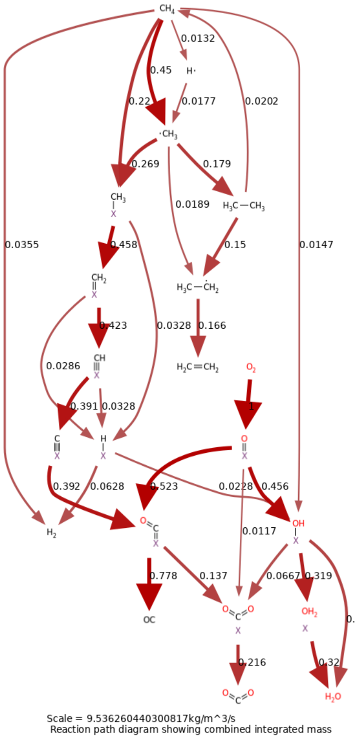

(b) Optimized LSR model reaction path

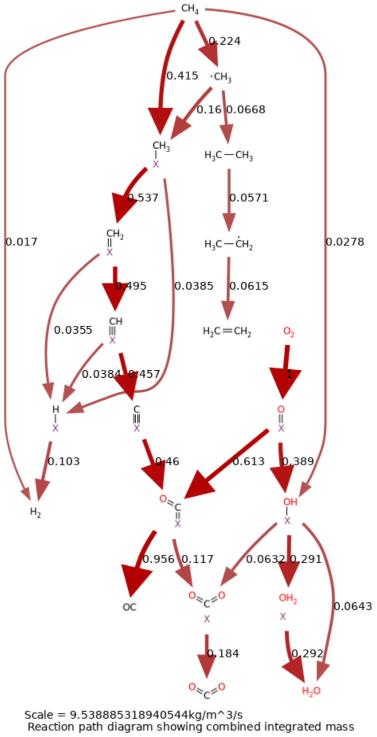

(c) Optimized ML model reaction path

Figure S2: Comparison of the reaction paths among Bayesian optimized LSR, DFT, ML models

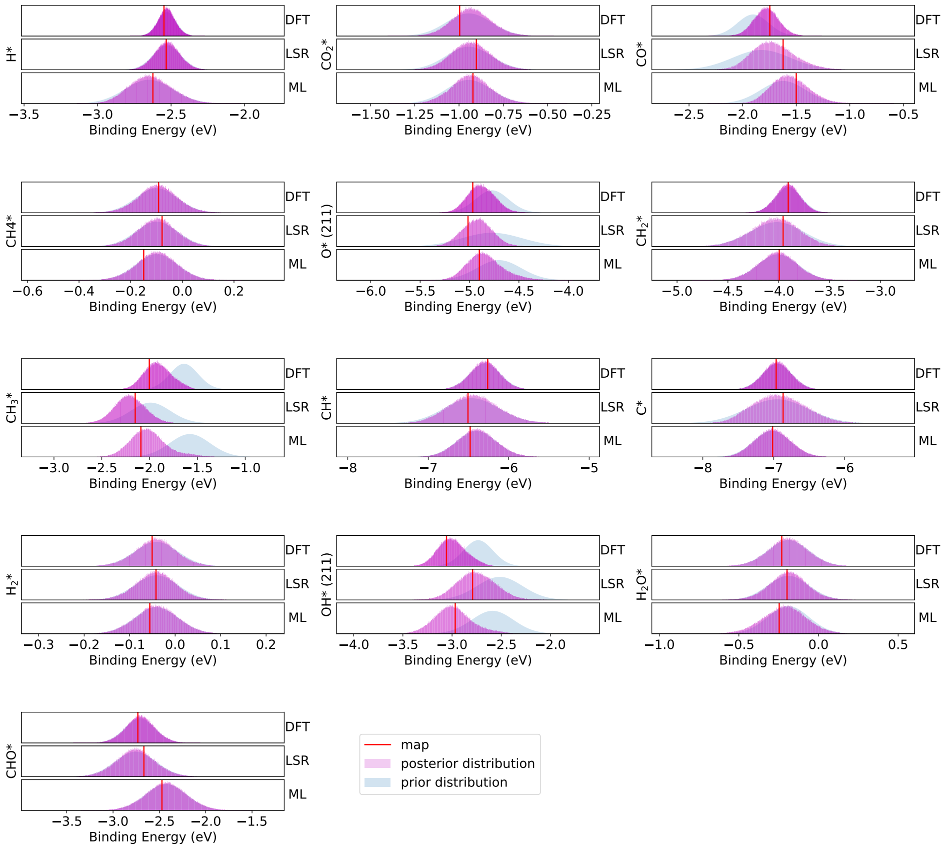

Figure S3: Prior distribution (blue shaded area), posterior distribution (pink shaded area) and the MAP value (red vertical line) for every adsorbed species in the model using the low experimental errors. O and OH were calculated on the 111 facet for this model.

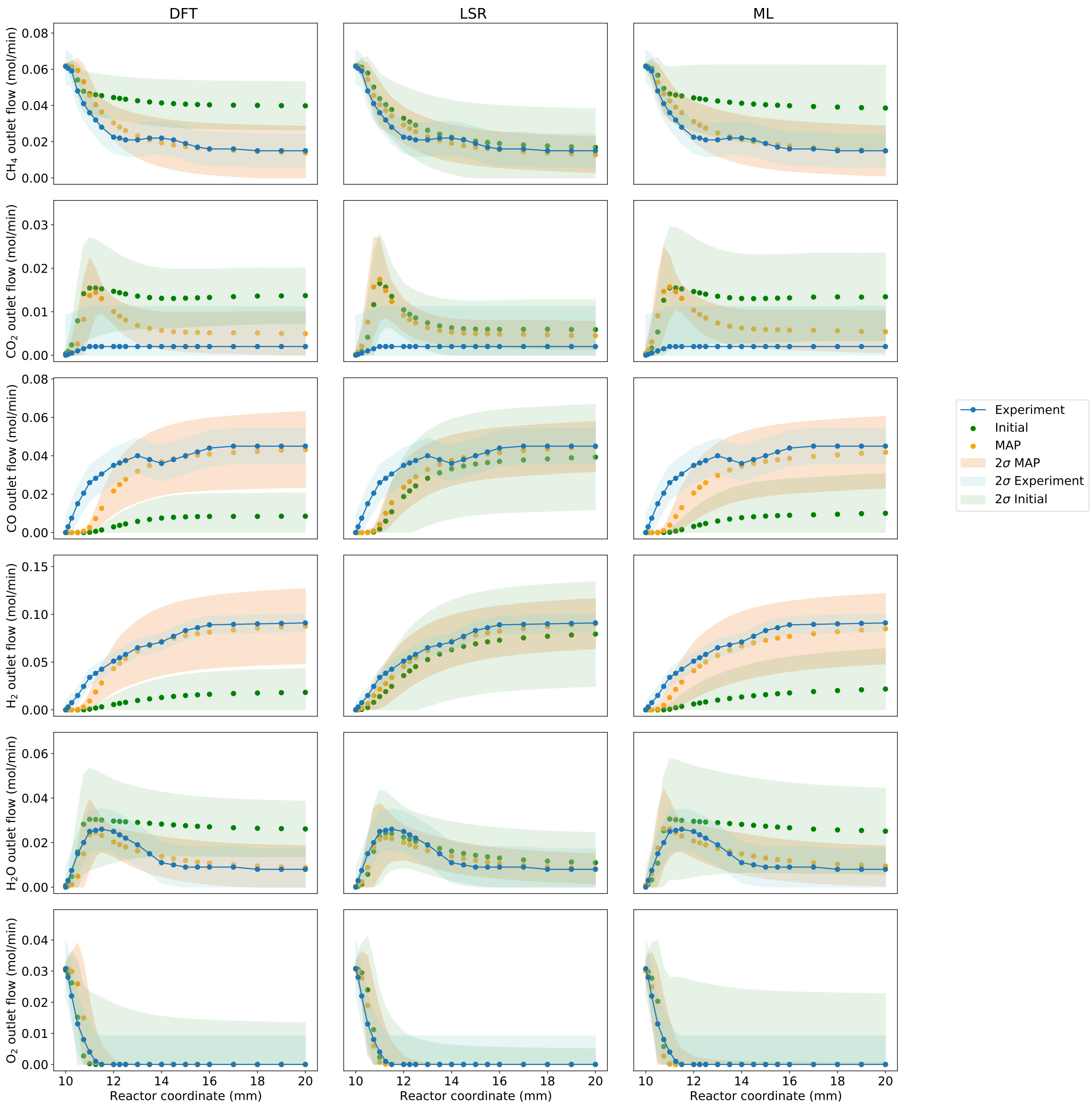

Figure S4: Gas phase flow rates observed in the unoptimized model using O and OH adsorbed on platinum 111 (green marker), the optimized model (orange marker), and the experimental data reported by Horn et al [S4]. The shaded regions are the 2 sigma (95% confidence) intervals for the uncertainty in the experimental data (light blue region) and the MAP outputs (light orange region).

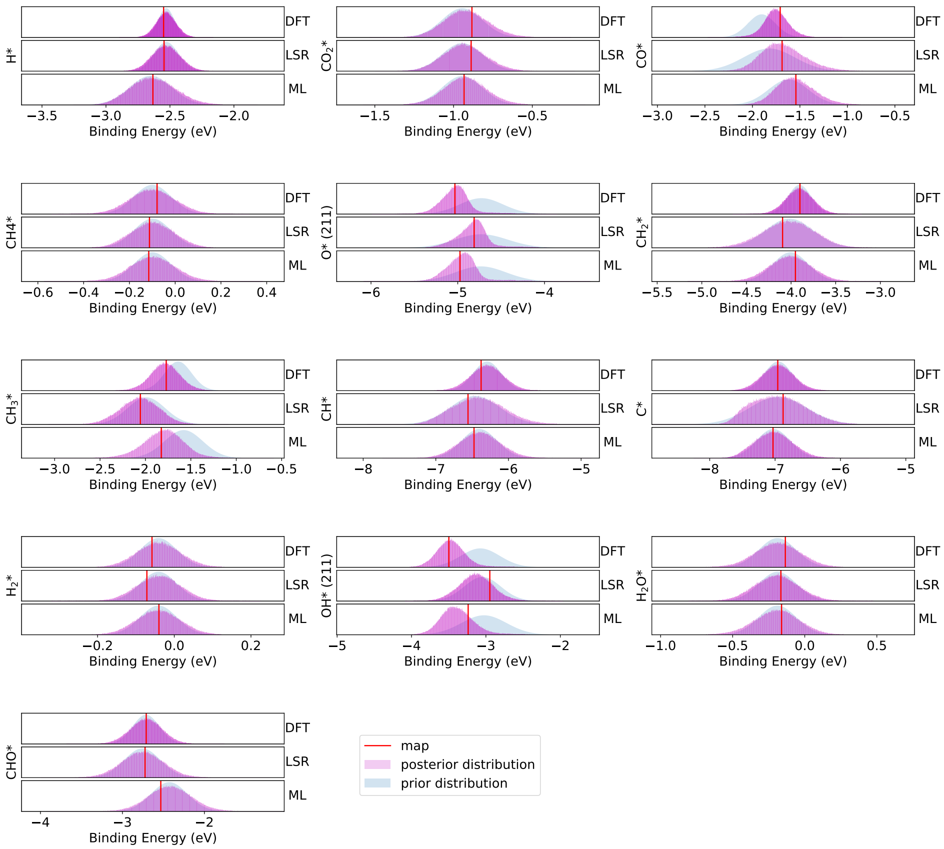

Figure S5: Prior distribution (blue shaded area), posterior distribution (pink shaded area) and the MAP value (red vertical line) for every adsorbed species in the model using the low experimental errors

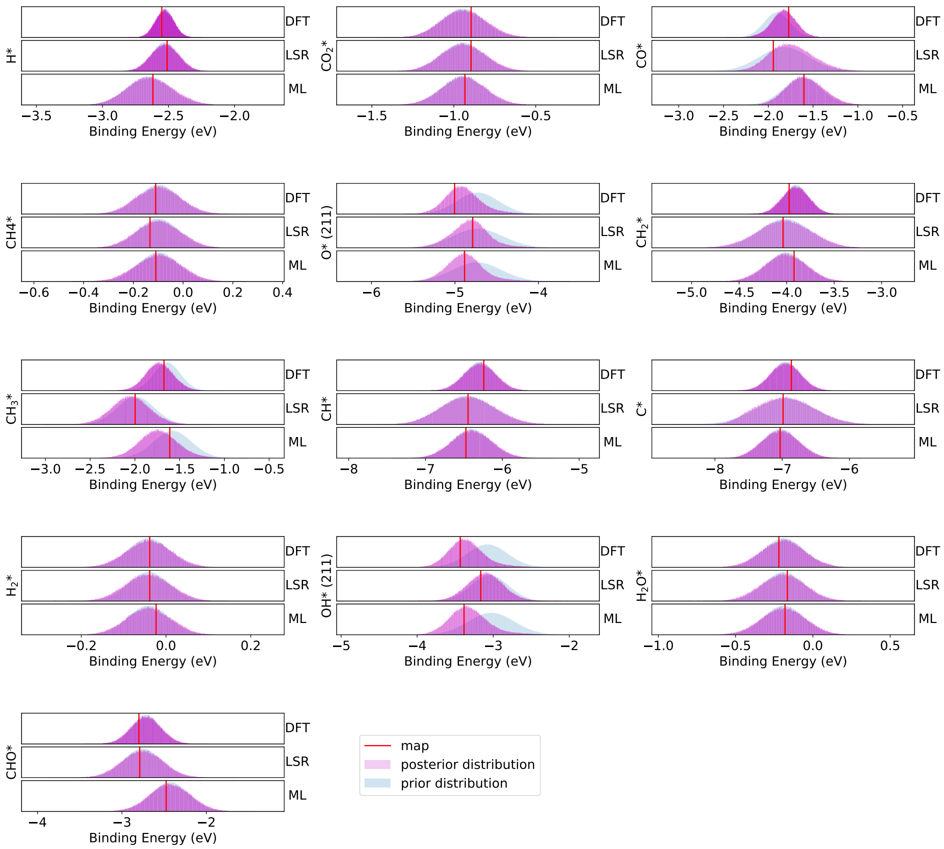

Figure S6: Prior distribution (blue shaded area), posterior distribution (pink shaded area) and the MAP value (red vertical line) for every adsorbed species in the model using the high experimental error range.

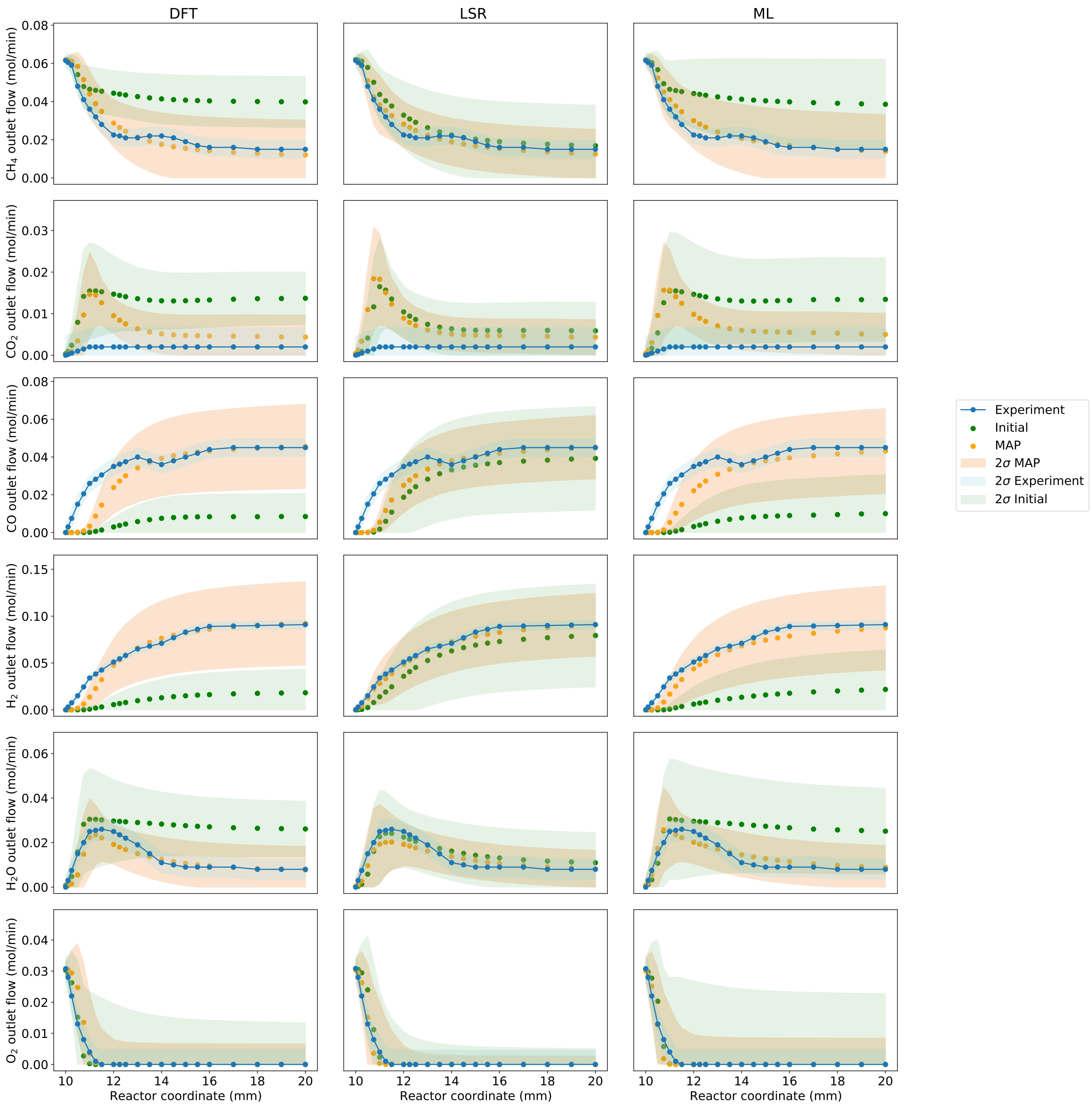

Figure S7: Gas phase flow rates observed in the unoptimized model (green marker), the optimized model using the low experimental error range (orange marker), and the experimental data reported by Horn et al [S4]. The shaded regions are the 2 sigma (95% confidence) intervals for the low error range uncertainty in the experimental data (light blue region) and the MAP outputs (light orange region)

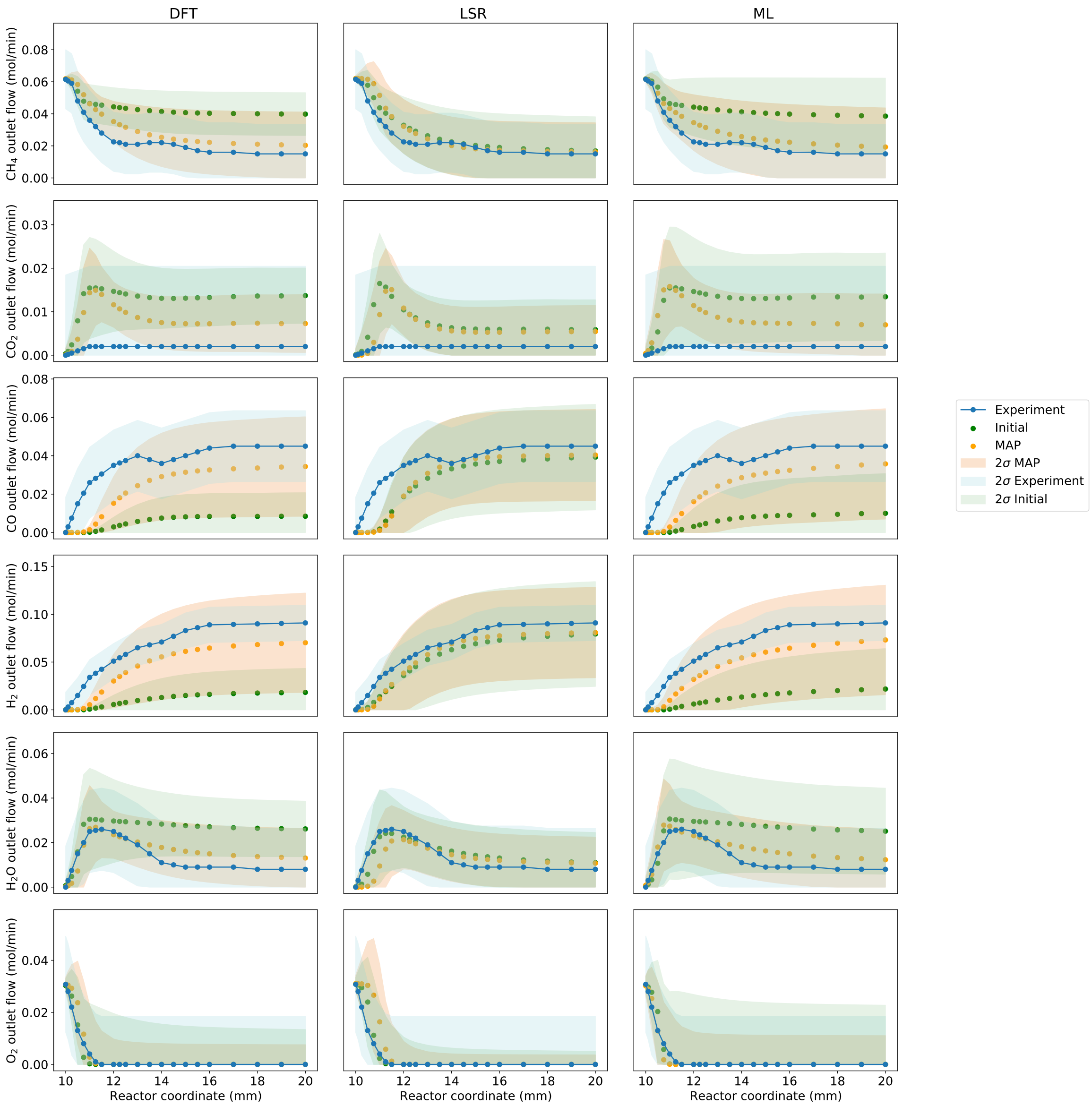

Figure S8: Gas phase flow rates observed in the unoptimized model (green marker), the optimized model using the high experimental error range (orange marker), and the experimental data reported by Horn et al [S4]. The shaded regions are the 2 sigma (95% confidence) intervals for the high error range uncertainty in the experimental data (light blue region) and the MAP outputs (light orange region)

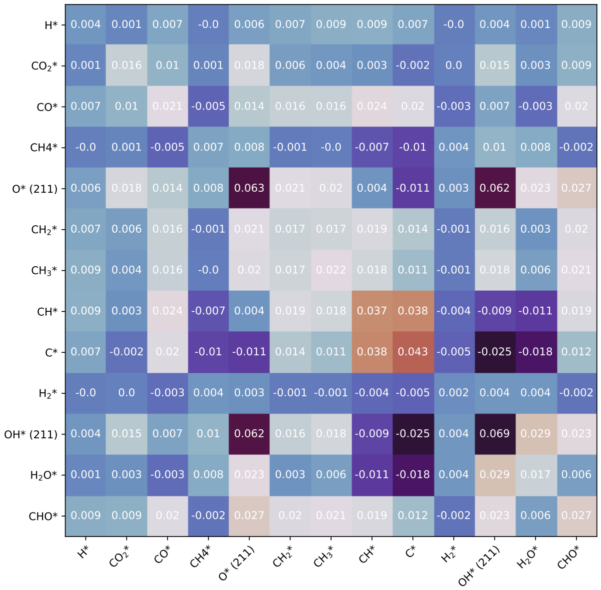

(a) prior covariance matrix for DFT model

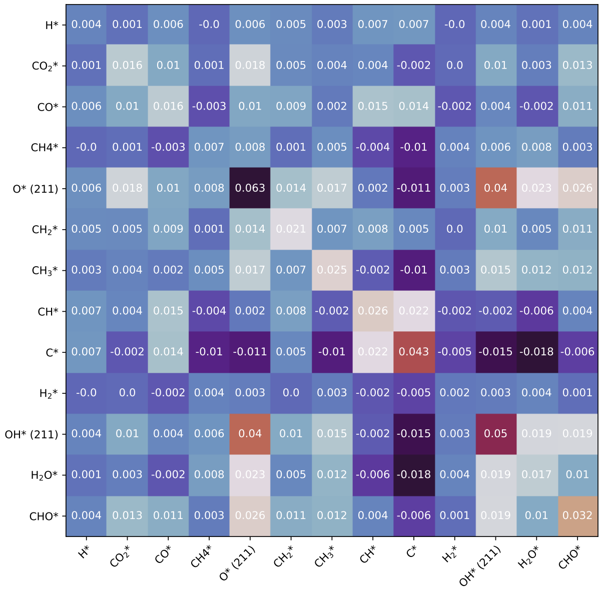

(b) prior covariance matrix for LSR model

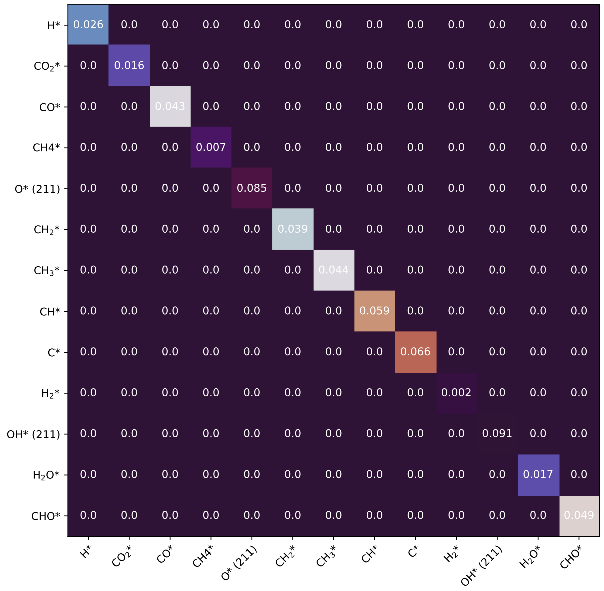

(c) prior covariance matrix for OCP model

Figure S9: Prior covariance matrices for DFT, LSR, and ML models

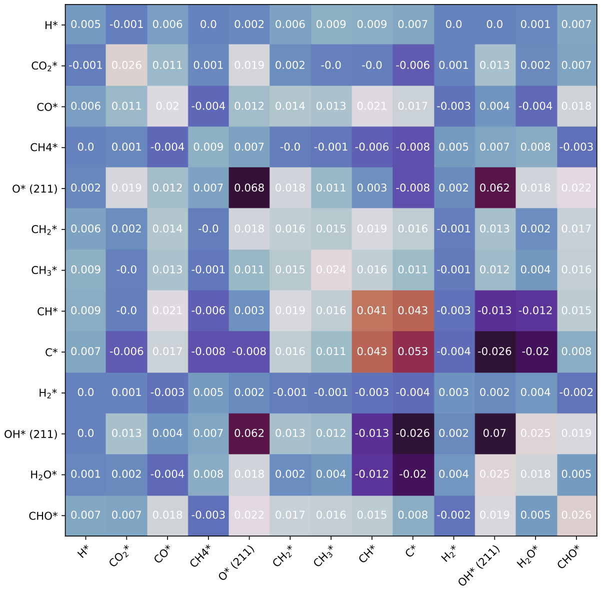

(a) prior covariance matrix for DFT model

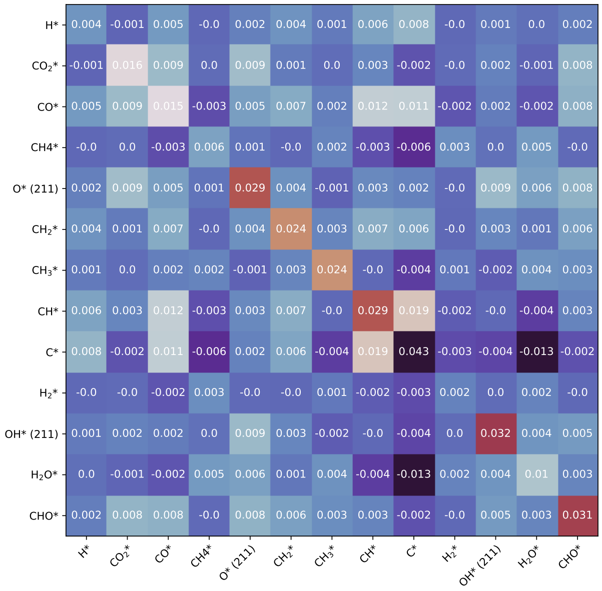

(b) prior covariance matrix for LSR model

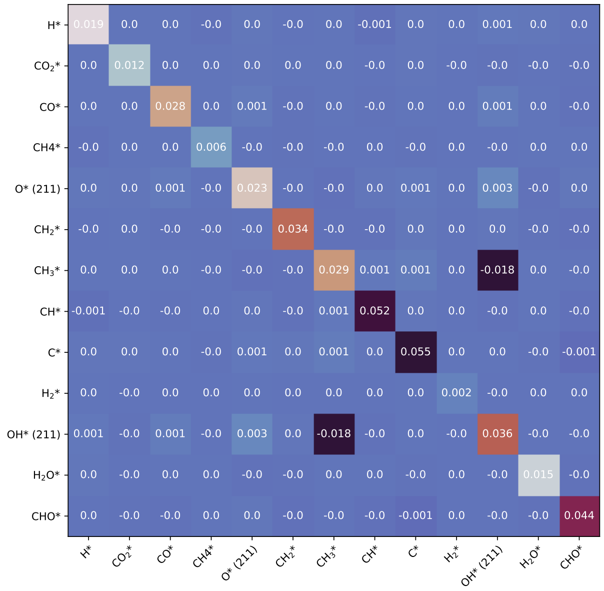

(c) prior covariance matrix for OCP model

Figure S10: Posterior covariance matrices for DFT, LSR, and ML models

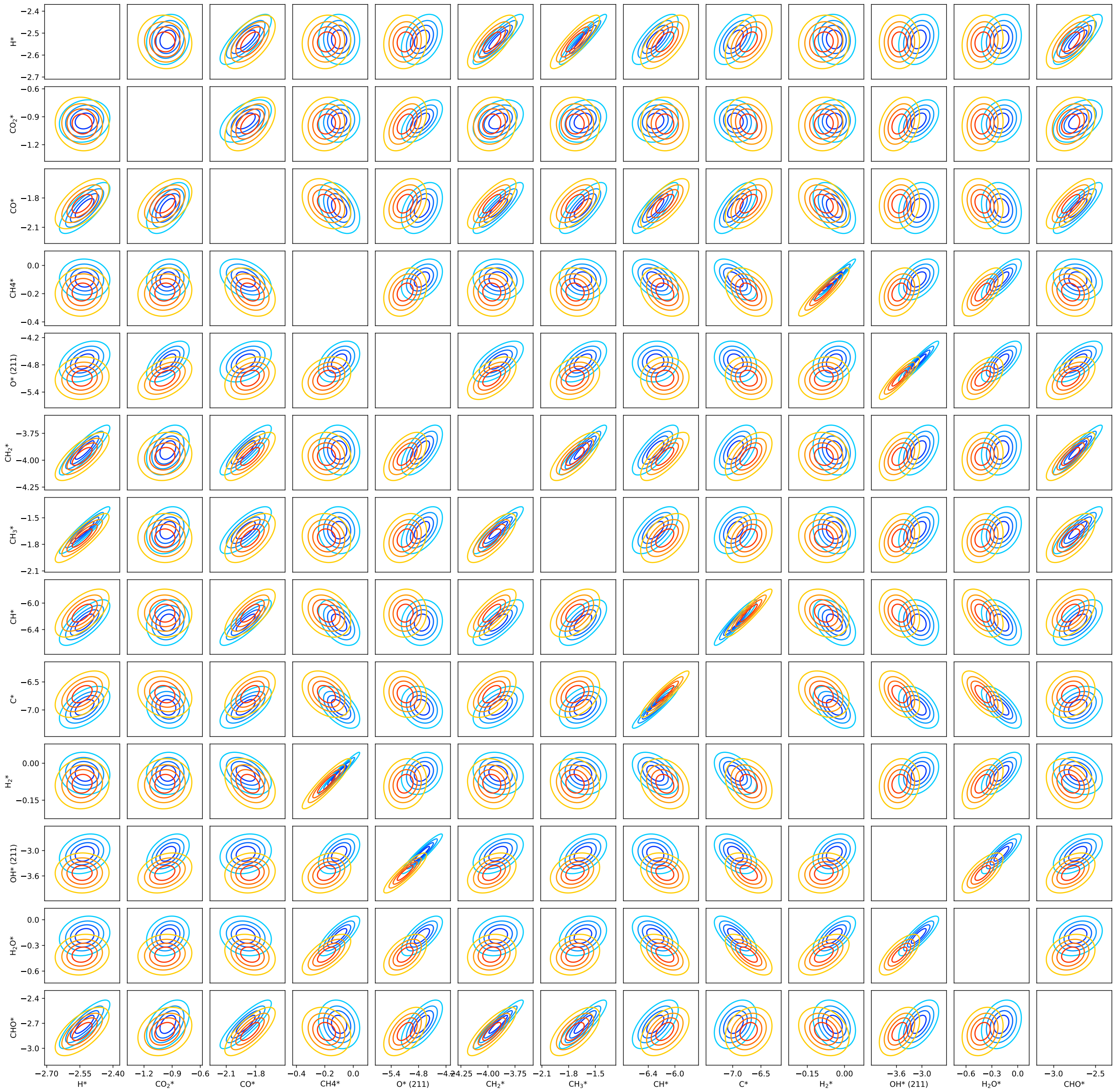

Figure S11: Posterior contour plots for DFT model using a prior covariance matrix obtained from the BEEF vdW ensembles for each species.

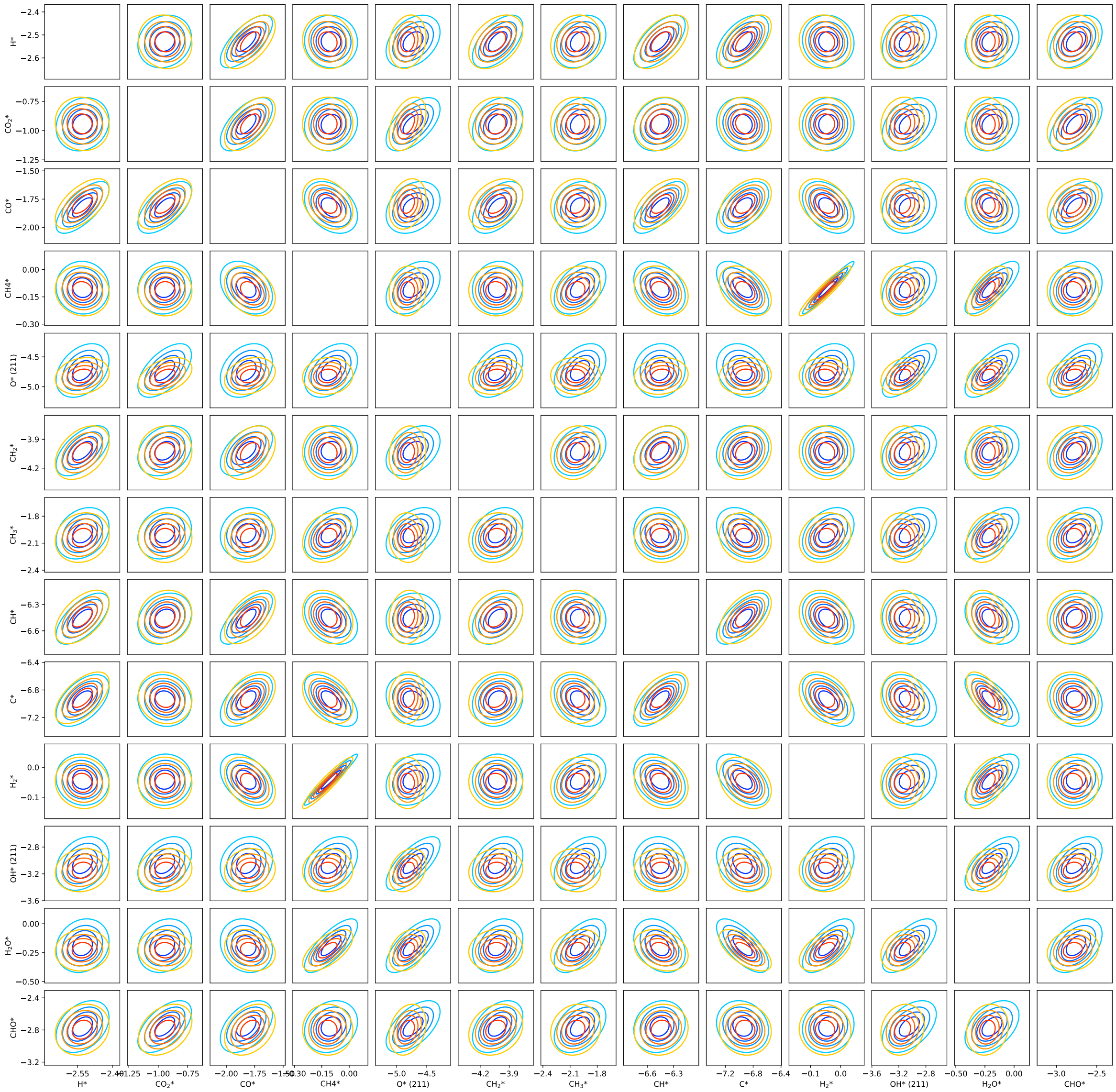

Figure S12: Posterior contour plots for LSR model using a prior covariance matrix obtained from the BEEF vdW ensembles for each species.

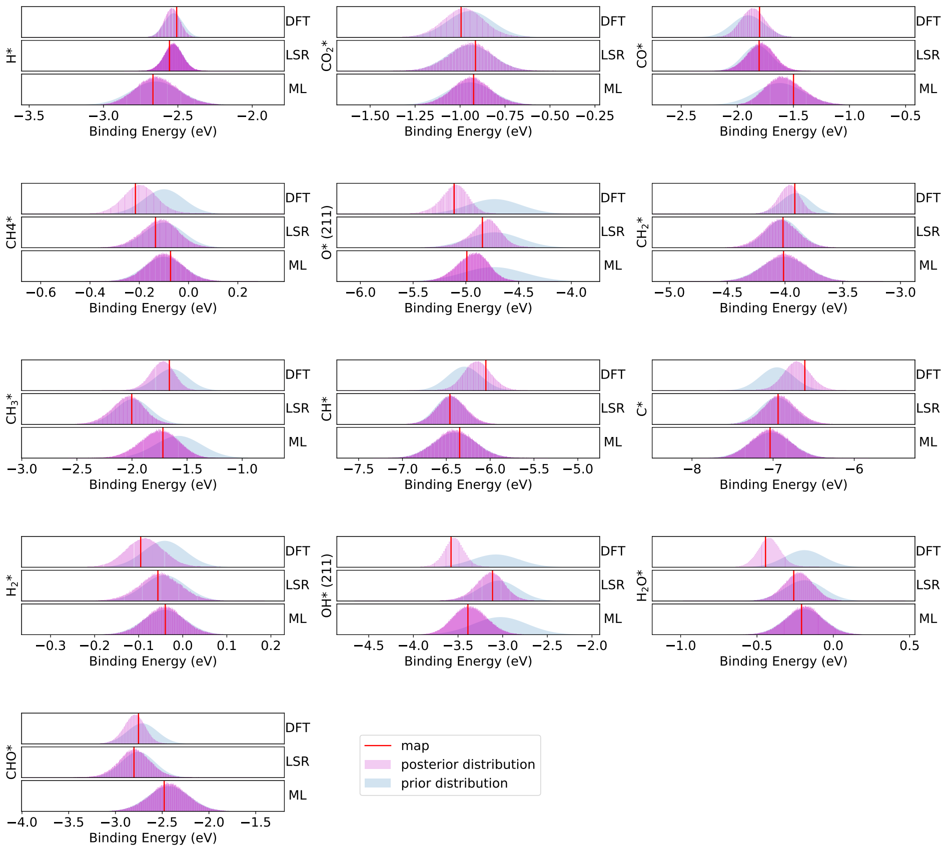

Figure S13: prior and posterior probability distributions for the runs with a prior covariance matrix.

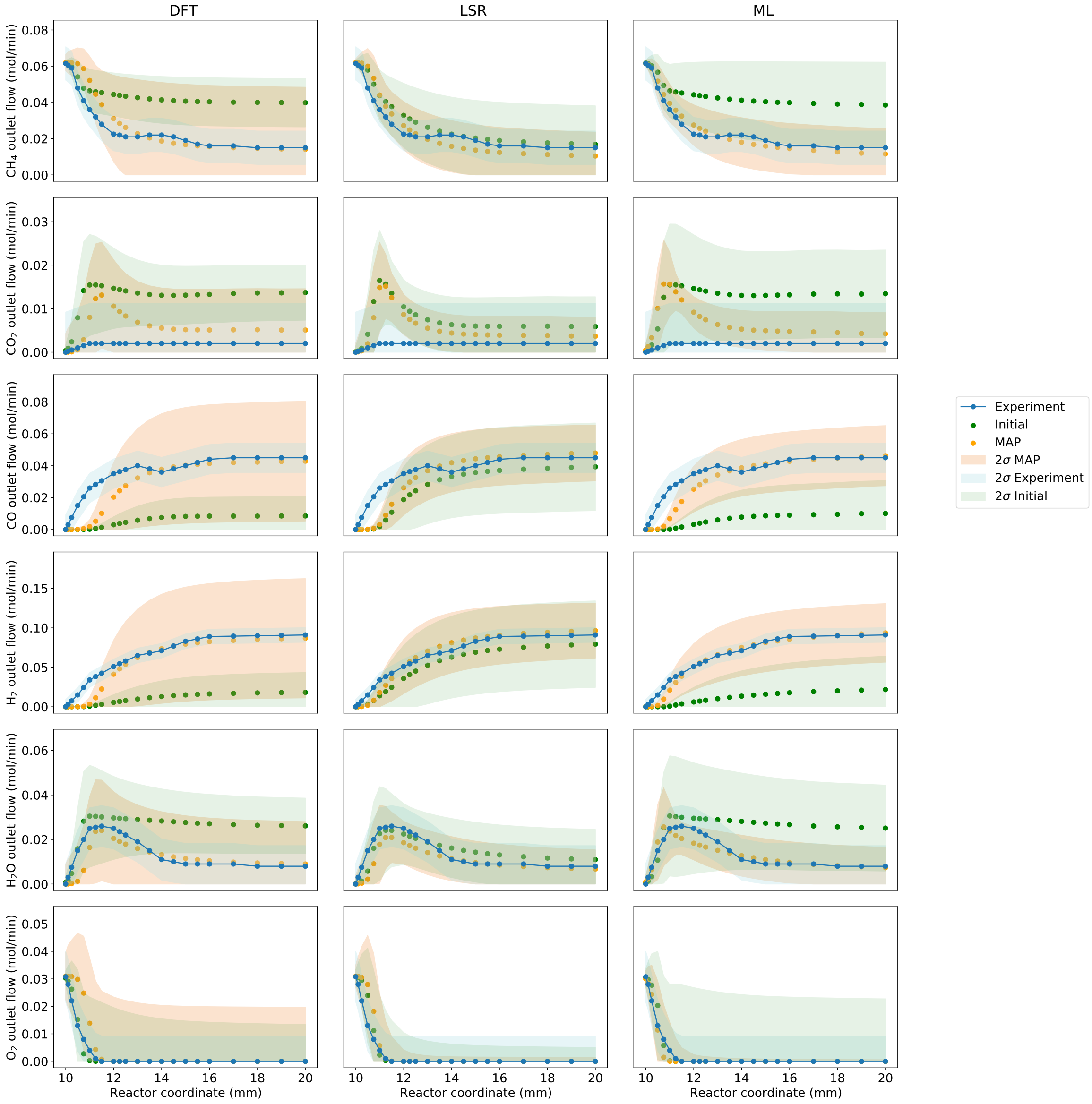

Figure S14: Output uncertainties for the runs involving covariance.

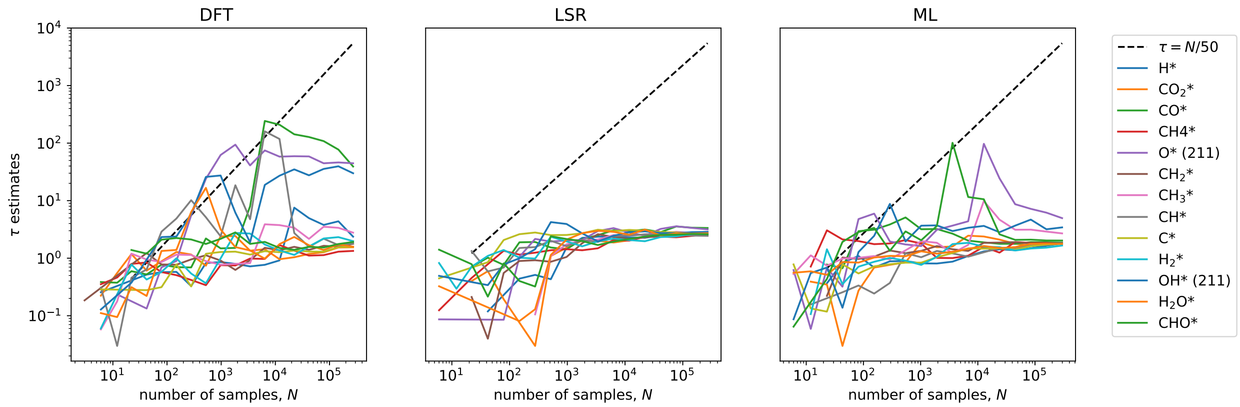

Figure S15: The autocorrelation time (ACT) for each species in the MCMC optimization for the models without covariance, and with O and OH adsorbed on the 211 facet.

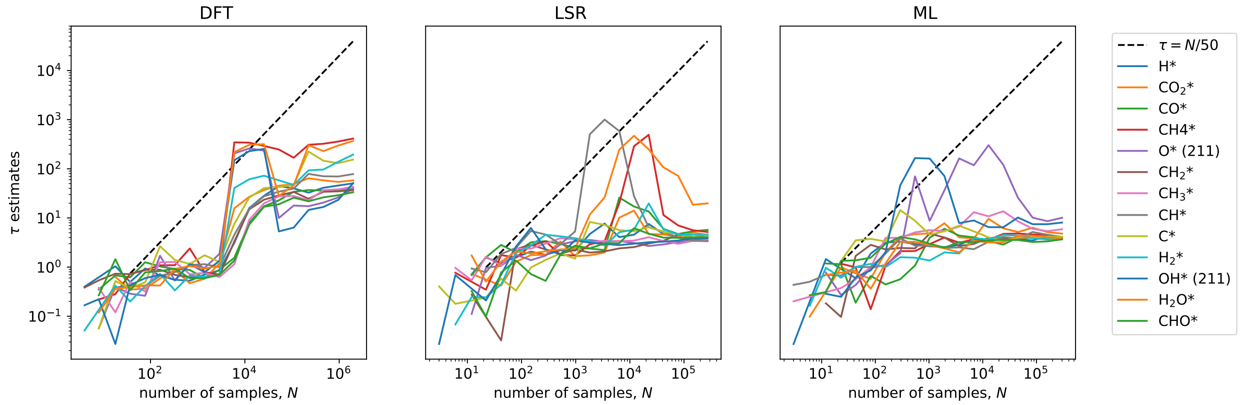

Figure S16: The autocorrelation time (ACT) for each species in the MCMC optimization for the models with covariance.

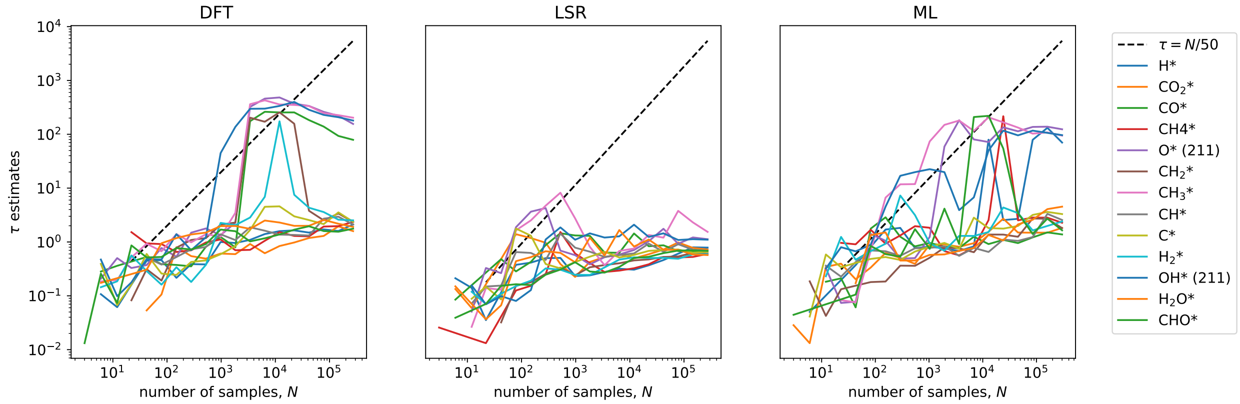

Figure S17: The autocorrelation time (ACT) for each species in the MCMC optimization for the model without covariance, and with O and OH adsorbed on the 111 facet.

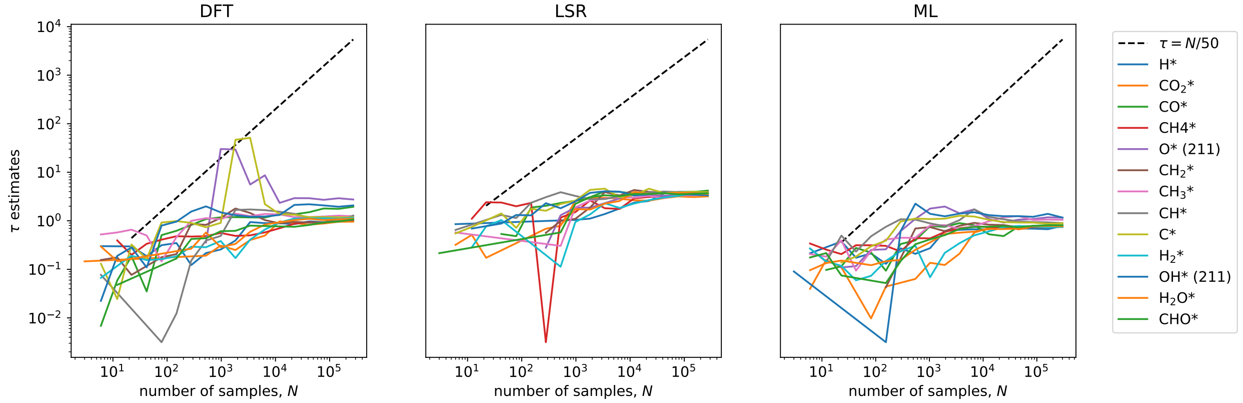

Figure S18: Covariance auto-correlation time (ACT) plots for the models using the low range experimental errors.

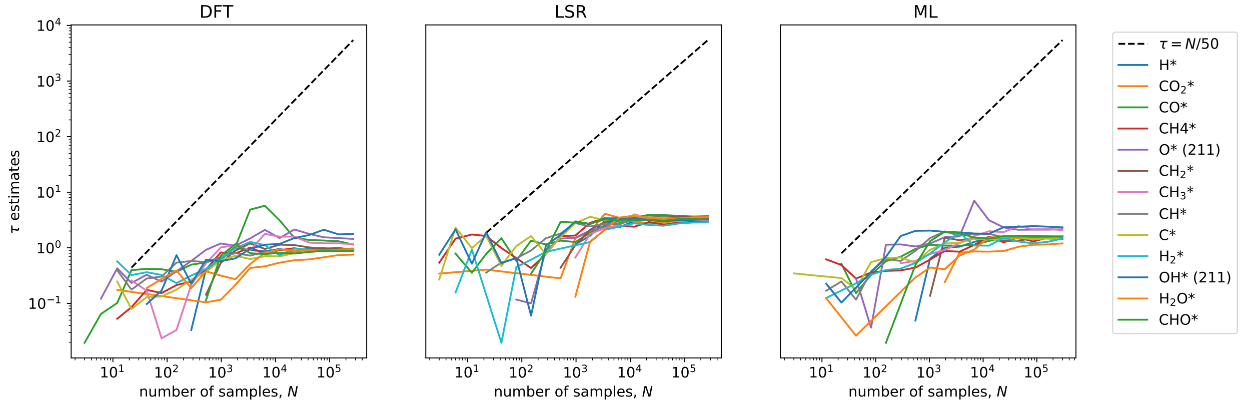

Figure S19: Covariance auto-correlation time (ACT) plots for the models using the high range experimental errors.

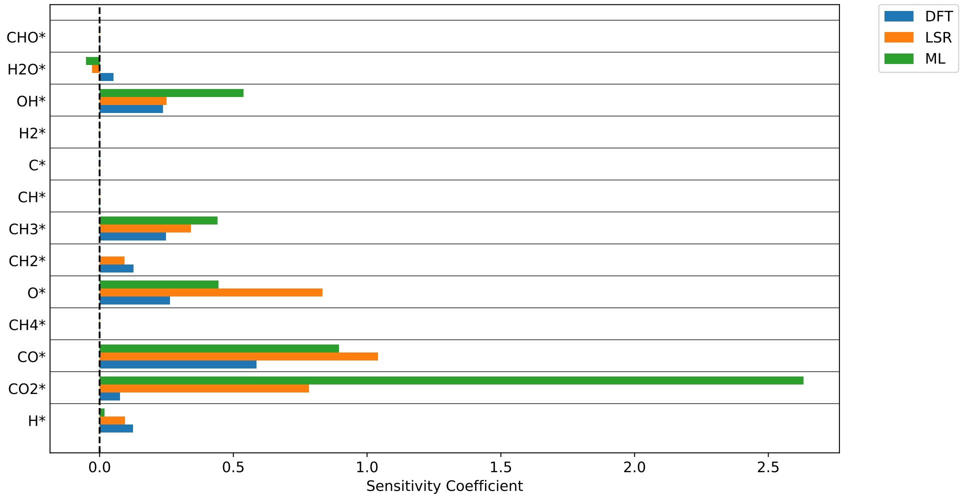

Figure S20: Sensitivity of the CO selectivity to each species binding energy.

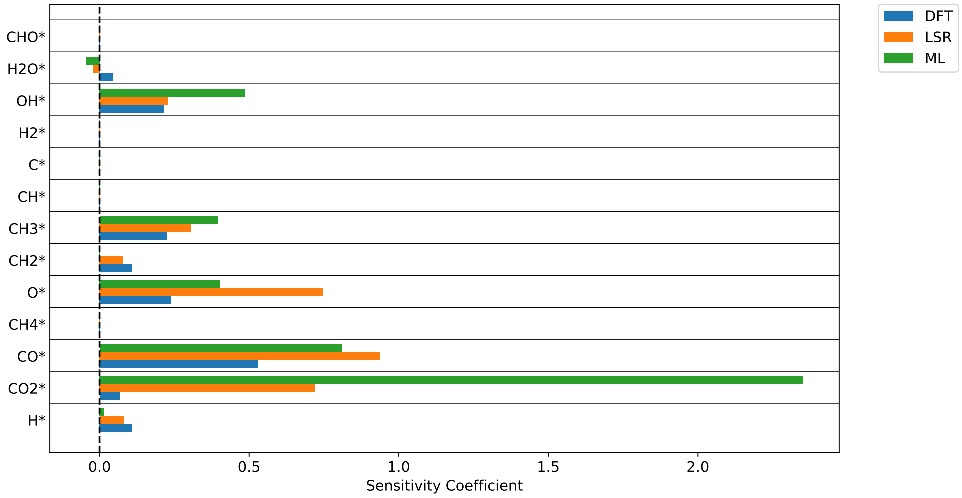

Figure S21: Sensitivity of the CO yield to each species binding energy.

0.7. Species Sensitivity Plots

The sensitivities of each species were analyzed for a number of benchmarks within the model, such as selectivity and yield for the expected products. The sensitivity of methane conversion can be found in the main paper, but the remaining plots can be found below in Figures S20 through S29.

One thing that may be noted, is that some models have inverse sensitivities to the other two models, for example the H_2O* binding energy has an inverse effect on the CH_4 conversion in the LSR and ML models, but not for the DFT model. This is likely an artifact of where the MAP model is in relation to the ”optimal” model, i.e. the model with the maximum possible value for the CH_4. Depending on which side of the maxima the MAP value falls, it will have either a positive or negative effect on the yield with an increase in the binding energy. this is more clearly illustrated in Figure S30, where we have indicated the MAP value for adsorbed water in each with a red dot.

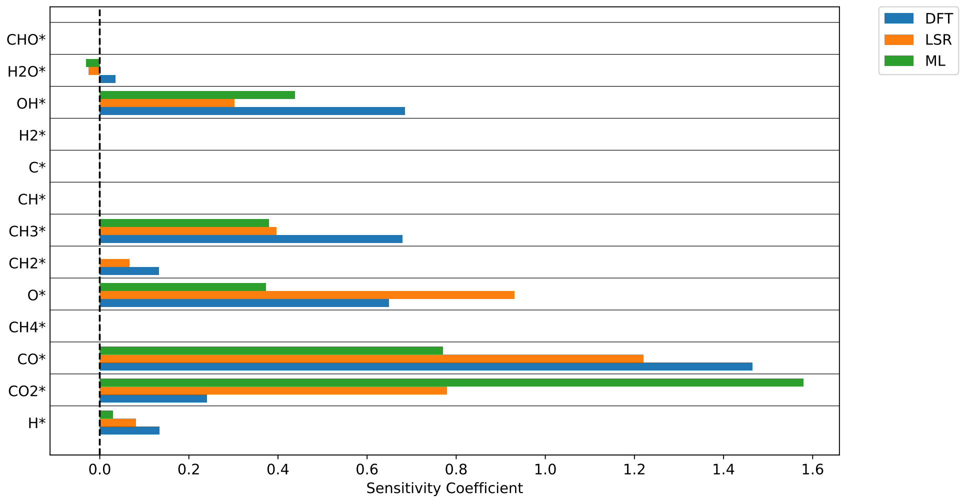

Figure S22: Sensitivity of the full oxidation selectivity to each species binding energy.

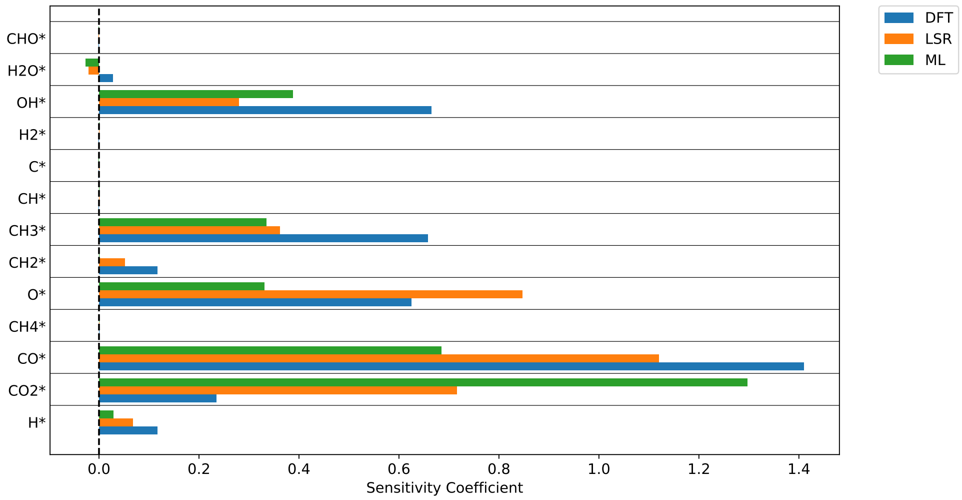

Figure S23: Sensitivity of the full oxidation yield to each species binding energy.

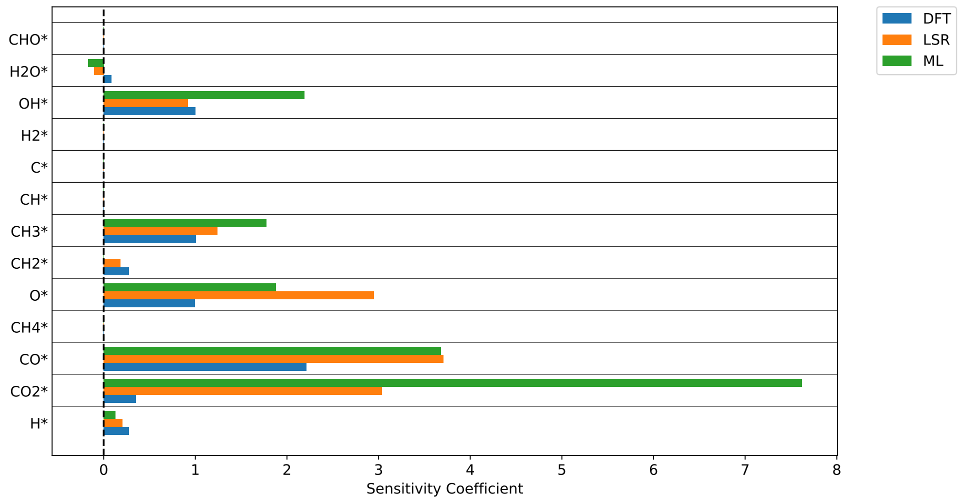

Figure S24: Sensitivity of the H_2 selectivity to each species binding energy.

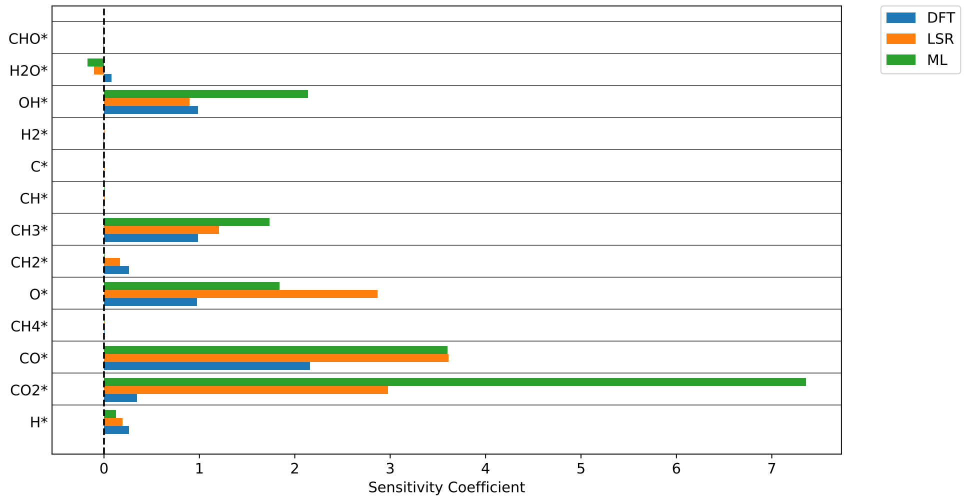

Figure S25: Sensitivity of the H_2 yield to each species binding energy.

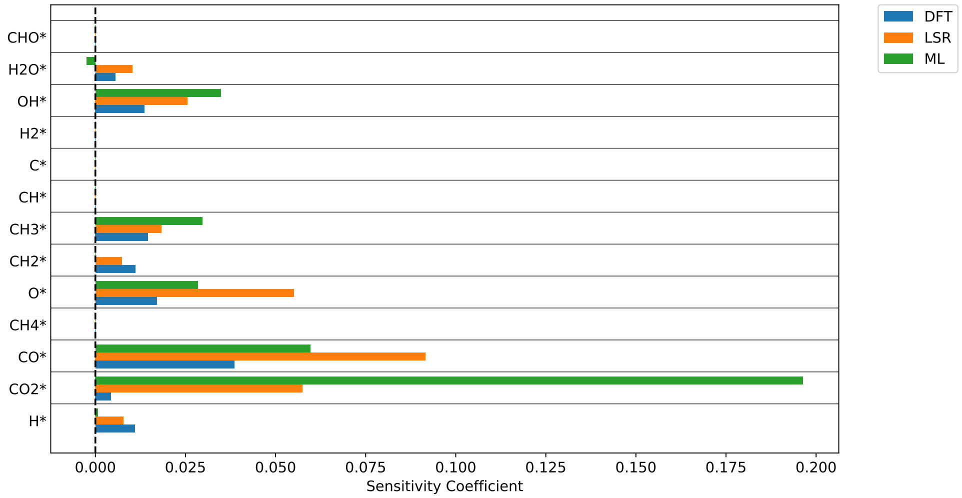

Figure S26: Sensitivity of the maximum CH_4 conversion to each species binding energy.

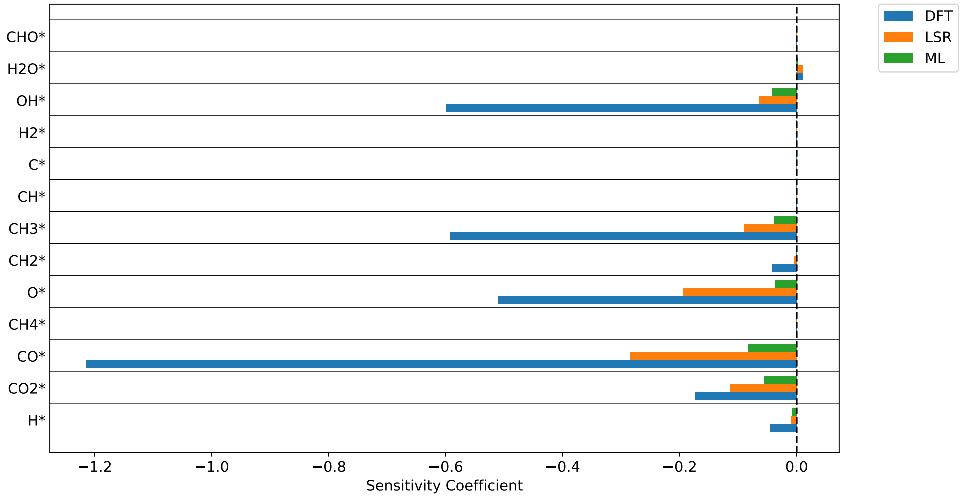

Figure S27: Sensitivity of the O_2 conversion to each species binding energy.

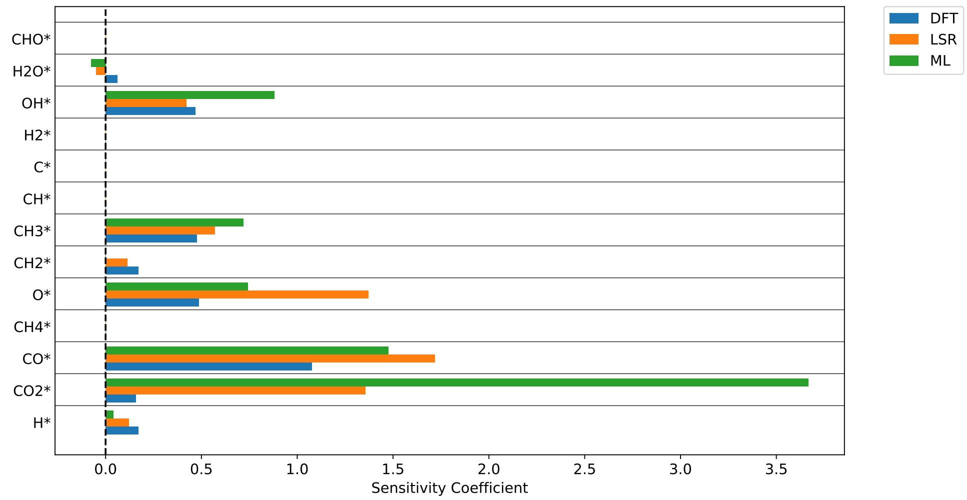

Figure S28: Sensitivity of the syngas selectivity to each species binding energy.

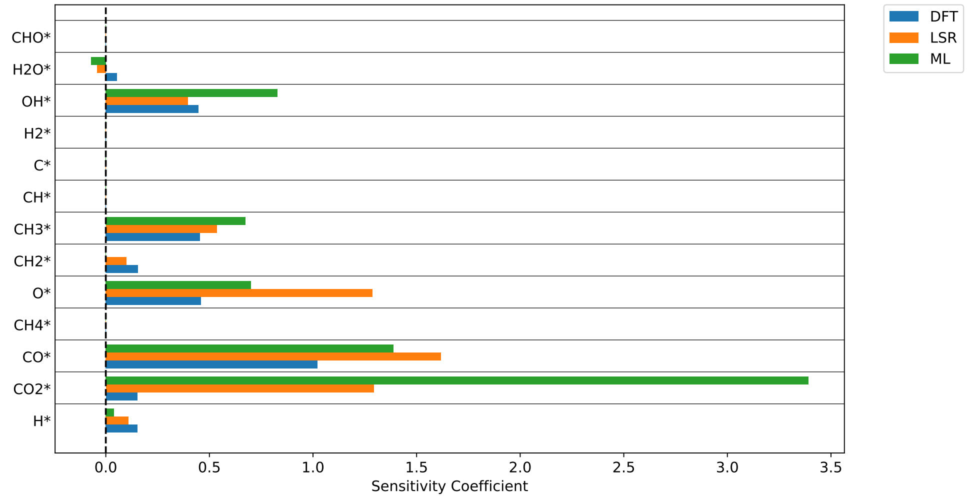

Figure S29: Sensitivity of the syngas yield to each species binding energy.

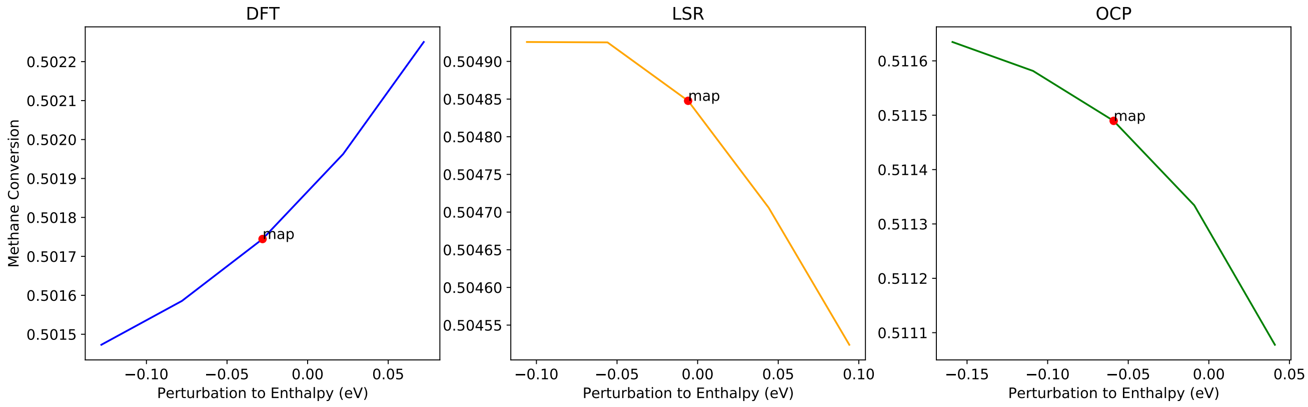

Figure S30: Comparison of where the MAP value for adsorbed water lies in relation to the local maxima for CH_4 yield.

species name	zero-point energy(eV)	DFT binding energy(eV)	vibrational frequencies(cm^{-1})
HRh	0.151	-2.68	690.5, 691.9, 1051.6
CORh	0.176	-1.62	79.5, 147.7, 265.3, 271.1, 324.1, 1747.6
ORh(211)	0.077	-4.92	291.5, 420.9, 534.8
CH_2Rh	0.642	-4.37	211.4, 393.1, 526, 527.1, 613.1, 799.4, 1342.4, 2932.7, 3008.9
CH_3Rh	0.899	-1.46	125.8, 214.9, 355.2, 374.8, 488.3, 501.1, 1192.6, 1342.8, 1346.3, 2812.7, 2872.7, 2876.8
CHRh	0.359	-6.34	380.6, 438.1, 563.2, 699.2, 704.1, 3006.6
CRh	0.087	-6.92	400.6, 499, 509.3
OHRh(211)	0.370	-3.22	119, 330.6, 411.7, 640.6, 658.7, 3663.5
CHORh	0.461	-2.65	146.4, 227.9, 246.9, 330.3, 466.4, 657.2, 1176.8, 1271.1, 2907.2
ORh	0.069	-4.96	303.0, 372.2, 445.2
OHRh	0.361	-2.87	119.0, 330.6, 411.7, 640.6, 658.7, 3663.5
CH_4Rh	1.224	-0.13	111.6i, 96.2i, 12.3i, 78.8, 126.6, 181.2, 1326.3, 1330.2, 1333.4, 1542.1, 1546.2, 2985.8, 3096.4, 3100.9, 3104.0
H_2ORh	0.634	-0.21	370.3i, 55.4i, 169.4, 284.7, 293.6, 329.2, 1578.0, 3721.2, 3851.6
H_2Rh	0.294	-0.058	20.7i, 32.0, 87.6, 95.8, 101.1, 4428.2
CO_2Rh	0.363	-0.17	31.2, 124.9, 163.0, 249.9, 301.9, 656.4, 670.4, 1318.6, 2336.9
CH_2Pt	0.683	-4.41	236.7, 416.9, 576.9, 675.3, 736.8, 911.4, 1378.2, 2995.4, 3084
CH_3Pt	0.954	-1.81	174.7i, 170.0i, 37.9, 502.3, 785.7, 785.8, 1211.2, 1431.3, 1431.7, 2998.9, 3104.4, 3104.8
CHOPt	0.505	-2.69	102.2, 208.5, 294.8, 341.7, 574.8, 831.6, 1201.7, 1736.3, 2859.7
CHPt	0.385	-6.37	456.2, 490.8, 626.1, 800.9, 804.4, 3038
COPt	0.2	-1.46	184.3, 223.5, 338.3, 340.2, 385.4, 1750.4
CPt	0.111	-6.72	439.9, 661, 687
HPt	0.131	-2.62	563.3, 565.4, 991.3
OHPt	0.352	-2.21	86.9, 182.5, 277.6, 505.2, 936.8, 3693.5
OHPt(211)	0.378	-2.97	151.5, 336.4, 412.3, 704.8, 810.3, 3687.1
OPt	0.072	-4.11	298.6, 421.6, 451.1
OPt(211)	0.076	-4.47	255.1, 446.7, 526.1

Table S2: DFT calculations, the DFT binding engery shown in the table is calculated by $\Delta H_{\mathrm{ads}}^{\mathrm{C_aO_bN_cH_d}*} = E_{\mathrm{DFT}}^{\mathrm{C_aO_bN_cH_d}*} - E_{\mathrm{DFT}}^{\mathrm{metal}} - E_{\mathrm{DFT}}^{\mathrm{C_aO_bN_cH_d(g)}}$

References

[S1] Filot, I.A.W., Broos, R.J.P., Van Rijn, J.P.M., Van Heugten, G.J.H.A., Van Santen, R.A., Hensen, E.J.M., 2015. First-Principles-Based Microkinetics Simulations of Synthesis Gas Conversion on a Stepped Rhodium Surface. ACS Catalysis 5, 5453–5467. URL: https://pubs.acs.org/doi/10.1021/acscatal.5b01391, doi:10.1021/acscatal.5b01391.

[S2] Foreman-Mackey, D., Hogg, D.W., Lang, D., Goodman, J., 2013. emcee: The mcmc hammer. PASP 125, 306–312. doi:10.1086/670067, arXiv:1202.3665.

[S3] Hickman, D.A., Schmidt, L.D., 1993. Steps in CH $_4$ oxidation on Pt and Rh surfaces: High-temperature reactor simulations. AIChE Journal 39, 1164–1177. URL: https://aiche.onlinelibrary.wiley.com/doi/10.1002/aic.690390708, doi:10.1002/aic.690390708.

[S4] Horn, R., Williams, K., Degenstein, N., Bitschlarsen, A., Dallenogare, D., Tupy, S., Schmidt, L., 2007. Methane catalytic partial oxidation on autothermal Rh and Pt foam catalysts: Oxidation and reforming zones, transport effects, and approach to thermodynamic equilibrium. Journal of Catalysis 249, 380–393. URL: https://linkinghub.elsevier.com/retrieve/pii/S0021951707001753, doi:10.1016/j.jcat.2007.05.011.

[S5] Karamanis, M., Beutler, F., Peacock, J.A., 2021. zeus: A Python implementation of Ensemble Slice Sampling for efficient Bayesian parameter inference. Monthly Notices of the Royal Astronomical Society 508, 3589–3603. URL: http://arxiv.org/abs/2105.03468, doi:10.1093/mnras/stab2867. arXiv:2105.03468 [astro-ph, physics:physics].

[S6] Kraus, P., Lindstedt, R.P., 2017. Microkinetic Mechanisms for Partial Oxidation of Methane over Platinum and Rhodium. The Journal of Physical Chemistry C 121, 9442–9453. URL: https://pubs.acs.org/doi/10.1021/acs.jpcc.7b02397, doi:10.1021/acs.jpcc.7b02397.
